# National and provincial impact and cost-effectiveness of *Haemophilus influenzae* type b conjugate vaccine in China: a modeling analysis

**DOI:** 10.1186/s12916-021-02049-7

**Published:** 2021-08-11

**Authors:** Haijun Zhang, Cristina Garcia, Wenzhou Yu, Maria Deloria Knoll, Xiaozhen Lai, Tingting Xu, Rize Jing, Ying Qin, Zundong Yin, Brian Wahl, Hai Fang

**Affiliations:** 1 China Center for Health Development Studies, Peking University, Beijing, China; 2 Department of Health Policy and Management, School of Public Health, Peking University, Beijing, China; 3 Department of International Health, Johns Hopkins Bloomberg School of Public Health, Baltimore, USA; 4 International Vaccine Access Center, Johns Hopkins Bloomberg School of Public Health, Baltimore, USA; 5 National Immunization Program, Chinese Center for Disease Control and Prevention, Beijing, China; 6 Department of Health Management and Policy, School of Public Health, Capital Medical University, Beijing, China; 7 Division of Infectious Diseases, Chinese Center for Disease Control and Prevention, Beijing, China; 8 Peking University Health Science Center, Chinese Center for Disease Control and Prevention Joint Research Center for Vaccine Economics, Beijing, China; 9 Key Laboratory of Reproductive Health, National Health Commission of the People’s Republic of China, Beijing, China

**Keywords:** Immunization, *Haemophilus influenzae* type b, China, Child health, Health economics

## Abstract

**Background:**

Globally, *Haemophilus influenzae* type b (Hib) vaccine has substantially reduced the burden of Hib invasive disease. However, China remains the only country not to include Hib vaccine into its national immunization program (NIP), although it accounts for 11% of global Hib deaths. We aimed to assess the cost-effectiveness of including Hib vaccine in China’s NIP at the national and provincial levels.

**Methods:**

Using a decision-tree Markov state transition model, we estimated the cost-effectiveness of Hib vaccine in the NIP compared to the *status quo* of Hib vaccine in the private market for the 2017 birth cohort. Treatment costs and vaccine program costs were calculated from Chinese Center for Disease Control and Prevention (CDC) and national insurance databases. Epidemiological data and other model parameters were obtained from published literature. Cases and deaths averted, quality-adjusted life years (QALYs) gained, and incremental cost-effectiveness ratios (ICER) were predicted by province. Deterministic and probabilistic sensitivity analyses were performed to explore model uncertainty.

**Results:**

Including Hib vaccine in the NIP was projected to prevent approximately 2700 deaths (93% reduction) and 235,700 cases of Hib disease (92% reduction) for the 2017 birth cohort at the national level. Hib vaccine was cost-effective nationally (US$ 8001 per QALY gained) compared to the GDP per capita and cost-effective in 15 of 31 provinces. One-way and scenario sensitivity analyses indicated results were robust when varying model parameters, and in probabilistic sensitivity analysis, Hib vaccine had a 64% probability of being cost-effective nationally.

**Conclusion:**

Introducing Hib vaccine in China’s NIP is cost-effective nationally and in many provinces. Less socioeconomically developed provinces with high Hib disease burden and low access to Hib vaccine in the current private market, such as those in the west region, would benefit the most from adding Hib vaccine to the NIP. In the absence of a national policy decision on Hib vaccine, this analysis provides evidence for provincial governments to include Hib vaccine into local immunization programs to substantially reduce disease burden and treatment costs.

**Supplementary Information:**

The online version contains supplementary material available at 10.1186/s12916-021-02049-7.

## Background

*Haemophilus influenzae* type b (Hib) is a common cause of pneumonia, meningitis, and other serious infections in children [1, 2]. China was estimated to be among the ten countries with the greatest number of Hib cases and deaths in children aged 1–59 months in 2000 [3]. Wahl et al. estimated that China still had approximately 3400 total Hib deaths in 2015 [4].

Vaccination is one of the most effective means of preventing Hib disease in a variety of settings around the world [5–7], and it remains an effective tool to reduce antibiotic resistance among some bacterial pathogens including Hib [8]. In September 2013, the World Health Organization (WHO) universally recommended the inclusion of Hib vaccines in all infant immunization programs worldwide, regardless of the availability of local or national surveillance data [2]. Hib vaccines have been introduced in 193 of 194 WHO member countries and regions [9]. China is the only WHO member that has not included Hib vaccine in its NIP. Rather, Hib vaccine is only available to children through the private market in China, where it first became available in 1996 [10]. While the global burden of Hib disease has decreased significantly in recent years with expanded access to Hib vaccine [11], China has a relatively large remaining burden of Hib disease [3, 4].

Policy decisions on introducing Hib vaccine into China’s NIP have been driven by uncertainties around the burden of Hib disease without national surveillance and the high price of Hib vaccines [10]. A new law enacted in 2019 empowering provincial public health officers to make their own policies regarding new vaccine introductions presents the opportunity for provinces to introduce Hib vaccine before it is nationally introduced into the NIP [12]. High-quality studies on the economic impact of Hib vaccination in China are limited, and subnational analyses are not available. National and provincial data on the economic impact of Hib vaccination in China are needed to inform policy decisions about expanding the use of Hib vaccines. To address this evidence gap, we evaluated the national and provincial cost-effectiveness of introducing Hib vaccine into China’s NIP compared to the *status quo* in the private market.

## Methods

### Model overview

**Fig. 1:**
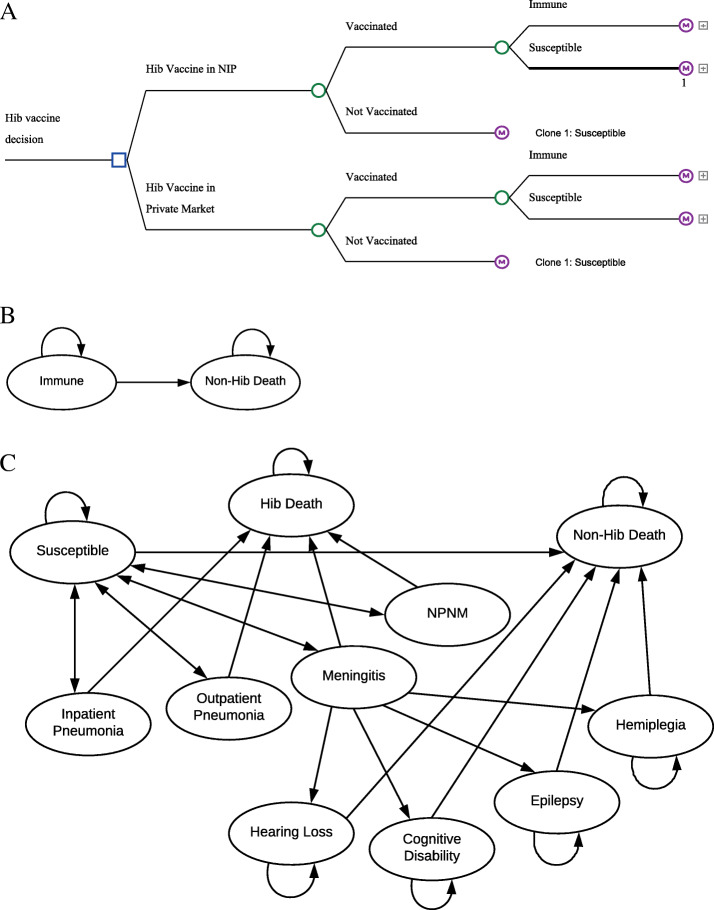
Markov decision tree for a single birth cohort comparing Hib vaccine in the national immunization program (NIP) vs. status quo (Hib vaccine in the private market). **A** Markov decision tree. **B** Hib Immune Markov diagram. **C** Hib susceptible Markov diagram

A decision-tree Markov state transition model was developed to estimate the impact of Hib vaccine on disease burden, quality-adjusted life years (QALYs), and costs for the 2017 birth cohort in each province in China (Fig. 1). The model compared Hib vaccine introduction into the NIP and the *status quo* where Hib vaccine is only available in the private market. The model tracked Hib pneumonia, meningitis, and non-pneumonia non-meningitis (NPNM) disease events over the cohort's first five years of life and estimated the QALYs gained over the life of the cohort. Children surviving past the neonatal period entered the model under both comparators (i.e., Hib vaccine in the NIP or *status quo*), and were assumed to be healthy but at risk of Hib infection depending on vaccination status (Fig. 1 and Additional file 1: Table S1).

The analysis was conducted from the societal perspective using a lifetime time horizon for the cohort. All costs and effects were discounted at 3% as recommended by the WHO [13], and all costs were converted to 2017 US dollars (1 US$ = 6.8 RMB), adjusting for inflation when necessary [14]. The model was developed using TreeAge Pro 2020 (TreeAge Software, Inc., Williamstown, MA). Results were estimated nationally and for the 31 provinces in mainland China and three geographically contiguous and socioeconomically distinct regions: east, central, and west according to National Bureau of Statistics of China. Parameter point estimates, plausibility ranges, and distributions are presented in Table 1.

**Table 1:** Province-specific model parameters and data sources

Model parameter	Base case value	Range (min and max)	Distribution	Source
**Population at risk and demographic parameters**				
Under-five mortality rate	Province-level data	Not varied	Beta	GBD study [15]
Neonatal mortality rate	Province-level data	Not varied	Not varied	Song et al. 2016 [16]
Child population	Province-level data	Not varied	Not varied	China National Bureau of Statistics and China CDC
**Epidemiologic data**				
Incidence of Hib meningitis (per 100,000 children 1–59 months)	Province-level data	Additional file 2: Table S2	Beta	Province-level disease burden model estimates
Incidence of Hib pneumonia (per 100,000 children 1–59 months)	Province-level data	Additional file 2: Table S2	Beta	Province-level disease burden model estimates
Incidence of Hib NPNM (per 100,000 children 1–59 months)	Province-level data	Additional file 2: Table S2	Beta	Province-level disease burden model estimates
Age distribution				
Hib pneumonia	0–11 months: 75% 12–23 months: 25% 24–59 months: 0%	Not varied	Not varied^*^	Watt et al. 2009 [3] and authors’ assumption
Hib meningitis and Hib NPNM	0–11 months: 73% 12–23 months: 21% 24–59 months: 6%	Not varied^*^	Not Varied^*^	Watt et al. 2009 [3] and authors’ assumption
Case fatality ratios				
Hib pneumonia	Province-level data	Additional file 2: Table S2	Beta	Province-level disease burden model estimates
Hib meningitis	Additional file 2	Additional file 2	Beta	Province-level disease burden model estimates
Hib NPNM	Additional file 2	Additional file 2	Beta	Province-level disease burden model estimates
Meningitis neurological sequelae	0.70%	0.53–0.88%	Beta	2018 China Health Statistics Yearbook [17]
Meningitis sequelae				
Probability of cognitive disability	1.60%	1.0–1.3%^†^	Beta	Edmond K et al. 2010 [18]
Probability of epilepsy	2.20%	2.1–3.2%^†^	Beta	Edmond K et al. 2010 [18]
Probability of hemiplegia	3.20%	2.2–8.1%^†^	Beta	Edmond K et al. 2010 [18]
Probability of hearing loss	4.60%	3.1–8.2%^†^	Beta	Edmond K et al. 2010 [18]
Probability of cochlear implant	40.00%	30.0–50.0%^†^	Beta	Sun B et al. 2015 [19]
**Vaccine efficacy and coverage**				
1-dose efficacy	60.00%	0–86.0%	Beta	Griffiths UK et al. 2012 and Watt et al. 2009 [3, 20]
2-dose efficacy	94.00%	69.0–98.0%	Beta	Griffiths UK et al. 2012 and Watt et al. 2009 [3, 20]
3-dose efficacy	95.00%	94.0–98.0%	Beta	Griffiths UK et al. 2012 and Watt et al. 2009 [3, 20]
4-dose efficacy	95.00%	94.0–98.0%	Beta	Assumed by 3-dose efficacy
Hib vaccine coverage in the private market	Province-level data	Additional file 4: Table S2	Triangular	China CDC
Hib vaccine coverage in a national immunization program	Region-level data	Additional file 4: Table S3	Triangular	Assumed by DTP vaccine coverage rate in China [16, 21]
**Cost of illness (USD)**				
Cost per inpatient pneumonia case	Province-level data	Additional file 3: Table S4	Gamma	CHIRA, China CDC and 2018 China Statistics Yearbook [22]
Cost per inpatient meningitis case	Province-level data	Additional file 3: Table S4	Gamma	CHIRA, China CDC and 2018 China Statistics Yearbook [22]
Cost per inpatient NPNM case	Province-level data	Additional file 3: Table S4	Gamma	CHIRA, China CDC and 2018 China Statistics Yearbook [22]
Cost per outpatient pneumonia case	Province-level data	Additional file 3: Table S4	Gamma	CHIRA, China CDC and 2018 China Statistics Yearbook [22]
Cost per cognitive disability case	1260	945–1575	Gamma	CHIRA
Cost per hearing loss case	3520	2640–4400	Gamma	CHIRA
Cost per epilepsy case	796	597–996	Gamma	CHIRA
Cost per hemiplegia case	1679	1259–2098	Gamma	CHIRA
Cost of cochlear implant per case	72,459	54,344–90,574	Gamma	Qiu J et al. 2017 [23]
Discounted cost of special education (age 6–18)	Province-level data	Additional file 3: Table S4	Gamma	2018 China Education Statistics Yearbook and 2018 China Education Expenditure Statistics Yearbook [24, 25]
Discounted lifetime productivity per capita	Province-level data	Additional file 3: Table S4	Gamma	2010 China census data [26]
**Utilities**				
Utility of meningitis	0.9768	0.5970–1	Beta	Bennett JE et al. 2010 [27]
Utility of outpatient pneumonia or NPNM	0.9963	0.9926–1	Beta	van Hoek AJ et al. 2012 [28]
Utility of inpatient pneumonia	0.9941	0.7948–1	Beta	Bennett JE et al. 2010 [27]
Utility of inpatient NPNM	0.9921	0.7825–1	Beta	Bennett JE et al. 2010 [27]
Utility of cognitive disability	0.62	0.51–0.73	Beta	Oostenbrink R et al 2002 [29]
Utility of hearing loss	0.91	0.83–1	Beta	Oostenbrink R et al 2002 [29]
Utility of epilepsy	0.83	0.75–0.91	Beta	Oostenbrink R et al 2002 [29]
Utility of hemiplegia	0.3903	0–1	Beta	Bennett JE et al. 2010 [27]
**Immunization costs (USD)**				
Vaccine price per dose	11.62	9.12–14.82	Gamma	China CDC
Cost of immunization delivery per dose	Province-level data	Additional file 4: Table S4	Gamma	China CDC [30]
Incidence of Hib vaccine severe adverse events (per 100,000 doses)	0.18	0–36.32	Beta	Li K et al. 2020 [31]
Cost of adverse events per case	903	677–1129	Gamma	2018 China Health Statistics Yearbook [32]
Wastage rate	5.00%	0.0–10.0%	Beta	Authors’ assumption

*NPNM* non-pneumonia non-meningitis, *GBD* Global Disease Burden, *CHIRA* Chinese Health Insurance Research Association

^*^Parameter not varied in the sensitivity analyses. Age distribution uncertainty is included in the disease burden incidence uncertainty.

^†^95% confidence interval (*CI*)

The base case values and ranges of all province-level data are presented in corresponding additional files

Additional file 1: Population at risk and demographic parameters

Additional file 2: Epidemiologic data

Additional file 3: Costs of Illnesses

Additional file 4: Hib vaccine coverage and immunization costs

### Epidemiological data

The province-specific probabilities of Hib severe and non-severe pneumonia, meningitis, and NPNM cases and deaths between ages 1 and 59 months were estimated from modeled provincial Hib incidence and mortality in 2017 [33] assuming no vaccination in the private market and following the Hib disease age distribution from published literature (Additional file 2: Table S2) [34]. We assumed all severe pneumonia, meningitis, and NPNM cases were hospitalized due to the severity of these syndromes, and all deaths occurred in hospitals. Access to care is high in most provinces, and most deaths of children under-five in China occur in healthcare facilities rather than in the community [1]. Children developing Hib meningitis in the model were at risk of long-term sequelae (i.e., cognitive disability, hearing loss, epilepsy, and hemiplegia) following probabilities from a global meta-analysis study [35].

### Disease burden costs

Provincial and regional costs of Hib disease were estimated using syndrome-specific data from published literature and health insurance data from the China Healthcare Insurance Research Association (CHIRA) comprising data from hospitals in all 31 provinces in mainland China between 2013 and 2017 [18]. The cost per inpatient and outpatient case of pneumonia, meningitis, and NPNM included direct medical, direct non-medical, and indirect costs. The cost of sequelae only included direct medical costs.

The average direct medical cost per case for each syndrome was estimated using the CHIRA individual-level medical cost data by ICD-10 code. Direct non-medical costs for inpatient pneumonia and meningitis, including the cost of transportation, accommodation, out-of-pocket medication, and other fees, were estimated from China CDC surveys conducted in 2015 in Gansu province [36] and 2014 in Shandong, Hebei, and Hubei provinces [37]. Estimates from these surveys were adjusted for each province using the ratio of the provincial total consumption expenditure obtained from the China Statistics Yearbook [22]. Because non-medical cost estimates for NPNM were not available, the cost was estimated using the average days hospitalized and the daily non-medical cost for inpatient pneumonia. Indirect costs associated with caregiver and visitor productivity loss and future lifetime productivity loss due to premature death and disability were estimated using the human capital approach. The methods used to estimate direct medical, non-medical costs, and indirect costs for each province are described in Additional file 3: Table S3 and S4.

### Vaccine efficacy and coverage rates

Because a uniform Hib vaccine schedule does not exist in China, the base case modeled a 3+1 dosing schedule assuming infants received primary doses by 6 months of age and a booster dose at the age of 18–24 months following the current schedule used in the private market and recommendations from the China CDC and vaccine manufacturers in China [38]. For the *status quo* scenario, provincial coverage rates for each dose of Hib vaccine in the private market were estimated by multiplying the total doses administered in each province from the China CDC by the distribution of children receiving 1, 2, 3, and 4 doses obtained from a 2019 facility-based survey of more than 6000 children in 10 provinces in China (Additional file 4: Table S2) [3, 39]. For coverage in the NIP, regional four-dose diphtheria-tetanus-pertussis vaccine (DTP) coverage was used as a proxy for Hib vaccine coverage because of the similar schedule in China. Dose-specific vaccine efficacies were estimated from a meta-analysis study of controlled clinical trials globally [20]. Due to the relatively low coverage of Hib vaccine currently in China and the wide dispersion of immunized children, herd immunity was not included in the base case, but was included in the sensitivity analysis.

### Vaccination costs

In China, several Hib vaccine products of varying prices are available in the private market. To estimate the cost of the vaccine in the private market, an average price per dose of US$ 11.6 (range US$ 9.1–14.9) was used based on centralized procurement data for seven Hib vaccine products in 2017 obtained from the China CDC [40]. For the base case analysis, the same vaccine price and schedule were used for both strategies to provide conservative results in the absence of guidance on vaccine introduction strategy from the Chinese government. The societal cost of the Hib vaccine program, including the governmental cost of routine immunization and the household cost of vaccine-seeking, was estimated using regional vaccine program data from a 2016 survey conducted by the China CDC in 15 provinces [30]. Governmental costs included the cost of vaccines, wastage, personnel, cold chain, surveillance, communication activities, training, and supervision at the national and provincial levels and the cost of serious adverse reaction. The vaccine-seeking costs included the cost of transportation and caregiver productivity loss. See Additional file 4 for the methods used to estimate the societal costs of the vaccine program for each strategy.

### Cost-effectiveness analysis

Incremental cost-effectiveness ratios (ICERs), defined as the incremental costs (i.e., Hib vaccine costs and disease costs) per Hib case averted, death averted and QALY gained, were used to compare the *status quo* and NIP strategies. QALY utilities were derived from published literature [27–29] and ranged from 0 to 1 where 0 represented death and 1 represented perfect health (Table 1). The Chinese government has no policy for assessing cost-effectiveness thresholds of vaccines, so the cost-effectiveness of Hib vaccine in the NIP was evaluated using two thresholds: (1) 2017 national and provincial GDP per capita thresholds as recommended by the Commission on Macroeconomics and Health [41]; and (2) thresholds estimated by Woods et al. for China (2017 US$ 1130 and US$ 4469), which account for the opportunity cost of the health expenditure and may be more appropriate to inform on resource allocation decisions [42]. The 2017 national GDP per capita was US$ 8774, and provincial GDPs are described in Additional file 1: Table S1 [22].

### Sensitivity analysis

Several sensitivity analyses were performed to test the robustness of model results and assess sources of model uncertainty. Deterministic sensitivity analyses (DSA) were conducted at the national level for all model parameters using the plausibility ranges specified in Table 1. For parameters with an unknown uncertainty range, the plausibility range was assumed to be 25% of the base value. Probabilistic sensitivity analysis (PSA) using Monte Carlo simulation (N=1000 iterations) was also done to assess the effects of changing multiple parameters simultaneously. Model uncertainty from the DSA and PSA were summarized using a tornado diagram and cost-effectiveness acceptability curves at the national level.

Scenario sensitivity analyses were performed to adjust the vaccine price per dose and vaccine schedules in the NIP and to include herd immunity. To estimate the influence of the NIP vaccine price on cost-effectiveness, we reduced the price of a Hib vaccine dose by 10–75% at national level. Although a 3+1 schedule is currently used in the private market, no decision has been made on the schedule for the NIP. To ensure the Chinese government has sufficient data to evaluate Hib vaccine introduction, we estimated the impact of a 3-dose NIP schedule on the cost-effectiveness.

The combined direct and herd immunity effects at different vaccine coverage levels were estimated using regression estimates previously published by Wahl et al. [4]. When Hib vaccine coverage is < 10% or ≥ 98%, the combined effect equals the direct effects. Because the assumed weighted vaccine coverage of all doses in the NIP strategy for all regions was more than 98%, herd immunity would not likely have any effect on the NIP strategy. However, with variable weighted vaccine coverage in the private market, herd immunity could increase the effectiveness of the *status quo* strategy in some provinces. See Additional file 4 for the regression model used as well as provincial and regional weighted vaccine coverage estimates [4].

## Results

### Impact of Hib vaccine

Access and coverage of Hib vaccine in the private market varied substantially by the province in China. National coverage was only 33%, ranging from over 50% in higher socioeconomically developed provinces like Shanghai and Tianjin to less than 5% in less socioeconomically developed provinces in the west region, like Tibet, Xinjiang, and Gansu.

The health effects and costs of introducing Hib vaccine into the NIP for the 2017 birth cohort are presented in Table 2. The model predicted that Hib vaccine in the NIP was projected to avert approximately 235,700 Hib cases and 2700 Hib deaths, a 93% reduction, over the first 5 years of life for the cohort. Most cases and deaths averted were pneumonia, with outpatient and inpatient pneumonia accounting for 80% and 17% of cases averted, respectively ( Additional file 5: Table S1). Guangdong and Hebei, the most populous provinces in China, had the greatest number of cases averted (i.e., > 10,000 cases). Most averted deaths were in Xinjiang and Yunnan provinces due to low Hib vaccine coverage and relatively high case fatality. The NIP strategy resulted in 85,388 QALYs gained over the cohort’s lifetime with most QALYs gained in provinces in the west and poorer provinces like Xinjiang and Yunnan.

**Table 2:** Estimated cases, deaths, and QALYs averted and ICERs over the first 5 years of life for the 2017 birth cohort by province for Hib vaccine in the NIP compared with Hib vaccine in the private market

Province and region	Status quo				NIP				Difference			ICERs			
	Total Hib cases	Total Hib deaths	QALYs	Total costs (US$)	Total Hib cases	Total Hib deaths	QALYs	Total costs (US$)	Cases averted	Deaths averted	QALYs gained	Incremental costs (US$)	US$ per case averted	US$ per death averted	US$ per QALY gained
Anhui	10,403	89	25,308,598	29,840,295	848	7	25,311,227	63,029,651	9555	82	2628	33,189,356	3474	405,945	12,628
Beijing	3629	16	6,992,155	11,325,844	307	1	6,992,620	23,483,721	3322	14	465	12,157,878	3660	842,025	26,151
Chongqing	4277	29	9,796,104	15,171,647	416	3	9,796,952	26,931,218	3862	27	848	11,759,571	3045	442,970	13,866
Fujian	7309	52	18,243,953	19,822,321	544	4	18,245,483	52,790,216	6765	48	1531	32,967,895	4873	688,732	21,535
Gansu	7372	138	10,572,870	18,650,750	420	8	10,577,033	27,428,442	6952	130	4163	8,777,692	1263	67,502	2,109
Guangdong	23,318	138	53,512,932	78,609,580	2390	14	53,516,894	145,501,435	20,928	123	3962	66,891,855	3196	542,026	16,881
Guangxi	10,967	99	23,942,820	29,549,022	845	8	23,945,699	70,637,008	10,123	91	2879	41,087,986	4059	450,407	14,269
Guizhou	8440	89	19,096,516	21,529,777	578	6	19,099,082	52,239,222	7862	83	2566	30,709,444	3906	370,831	11,968
Hainan	2165	47	4,153,964	8,673,501	161	4	4,155,318	12,362,132	2004	44	1354	3,688,631	1840	84,335	2725
Hebei	18,251	200	29,499,659	40,852,608	1255	14	29,505,827	71,021,772	16,996	186	6168	30,169,164	1775	161,982	4892
Heilongjiang	4334	31	6,359,528	7,395,149	334	2	6,360,481	18,366,561	4000	29	953	10,971,412	2743	378,325	11,515
Henan	14,483	124	45,111,621	56,142,230	1292	11	45,115,341	113,686,313	13,191	113	3720	57,544,084	4362	508,959	15,469
Hubei	6316	41	20,415,487	26,105,748	658	4	20,416,654	51,335,337	5658	37	1167	25,229,589	4459	681,881	21,623
Hunan	11,281	56	28,368,463	24,041,243	821	4	28,370,122	73,935,365	10,461	52	1660	49,894,122	4770	956,977	30,065
Inner Mongolia	5367	64	6,487,144	10,913,451	323	4	6,489,099	17,991,833	5045	60	1955	7,078,383	1403	117,771	3,620
Jiangsu	13,746	35	25,094,846	18,744,397	841	2	25,095,960	75,413,883	12,904	33	1114	56,669,485	4391	1,709,314	50,853
Jiangxi	8158	160	18,148,883	31,690,026	684	13	18,153,489	45,222,379	7473	146	4606	13,532,353	1811	92,556	2938
Jilin	4495	38	5,115,023	6,981,473	309	3	5,116,189	13,080,444	4186	35	1166	6,098,970	1457	174,082	5231
Liaoning	6451	17	9,158,027	6,996,751	445	1	9,158,562	26,430,504	6006	16	535	19,433,753	3236	1,233,132	36,353
Ningxia	1979	37	2,943,166	4,797,602	114	2	2,944,256	8,165,032	1865	35	1090	3,367,430	1806	96,869	3091
Qinghai	2002	63	2,458,854	7,477,517	113	4	2,460,726	7,028,785	1889	60	1872	− 448,732	Cost-saving	Cost-saving	Cost-saving
Shaanxi	7748	135	13,716,704	22,367,095	489	9	13,720,747	37,958,606	7260	126	4042	15,591,511	2148	123,326	3857
Shandong	15,018	49	47,123,397	41,661,088	1185	4	47,124,958	118,500,002	13,833	46	1561	76,838,914	5555	1,687,784	49,231
Shanghai	1568	7	6,438,987	14,263,336	300	1	6,439,171	18,708,669	1268	6	184	4,445,332	3506	776,871	24,137
Shanxi	7610	115	11,724,642	16,858,840	479	7	11,728,100	29,745,170	7130	107	3458	12,886,331	1807	120,112	3726
Sichuan	12,540	165	26,965,857	49,556,572	1294	17	26,970,457	73,280,239	11,246	148	4600	23,723,667	2110	160,170	5157
Tianjin	1532	11	3,616,534	6,694,387	176	1	3,616,852	10,604,944	1356	10	318	3,910,557	2885	391,035	12,278
Tibet	1794	85	1,764,971	8,508,444	98	5	1,767,356	5,253,368	1695	80	2385	− 3,255,076	Cost-saving	Cost-saving	Cost-saving
Xinjiang	9254	414	12,338,924	42,483,700	506	23	12,350,709	35,655,488	8748	391	11,785	− 6,828,212	Cost-saving	Cost-saving	Cost-saving
Yunnan	14,896	336	19,920,852	49,455,885	1045	24	19,930,438	56,345,008	13,851	312	9586	6,889,123	497	22,055	719
Zhejiang	9079	36	21,204,981	32,599,391	852	3	212,06,047	60,805,003	8228	32	1066	28,205,612	3428	872,003	26,459
East	102,064	608	225,039,435	280,243,205	8455	50	225,057,693	615,622,281	93,609	558	18,258	335,379,076	3583	600,809	18,369
Central	67,079	653	160,552,246	199,055,004	5425	52	160,571,603	408,401,221	61,654	601	19,358	209,346,217	3395	348,048	10,815
West	86,639	1654	150,004,782	280,461,461	6240	110	150,052,554	418,914,249	80,398	1544	47,772	138,452,788	1722	89,674	2898
National	255,781	2915	535,596,462	759,759,670	20,122	212	535,681,850	1,442,937,751	235,659	2704	85,388	683,178,081	2899	252,686	8001

Rows and columns may not sum to the total due to rounding

Introducing Hib vaccine into the NIP was estimated to cost US$ 1.4 billion (US$ 1.1 billion to the Chinese government) in vaccine procurement, programmatic costs, and indirect costs (Table 2 and Additional file 5: Table S2). However, investment in vaccination would be partially offset by savings of US$ 377 million from averted treatment costs and increased lifetime productivity nationally.

### Cost-effectiveness analysis

The national ICERs per case averted, per death averted, and per QALY gained were US$ 2899, US $252,686, and US$ 8001, respectively. ICERs per QALY gained were less than the national GDP per capita (US$ 8774) in 2017, indicating Hib vaccine in the NIP was highly cost-effective.

**Fig. 2:**
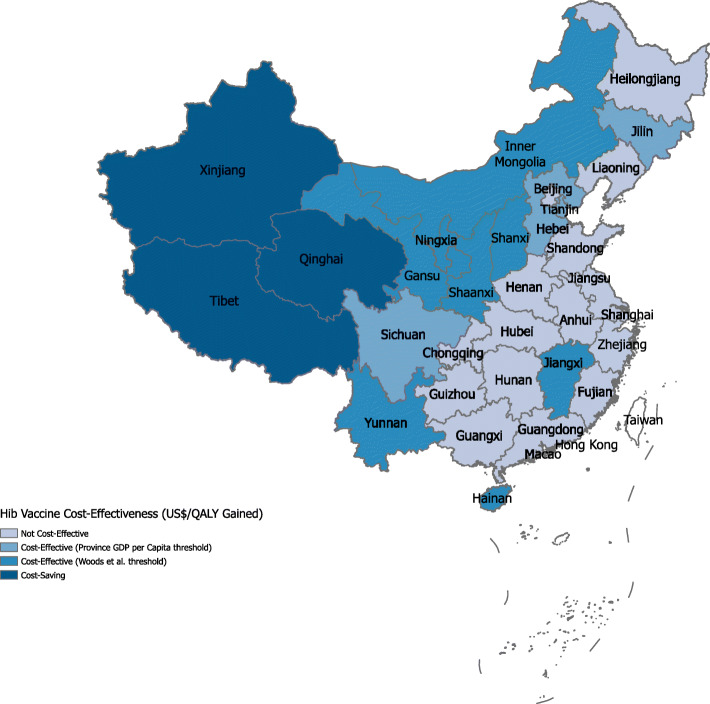
Cost-effectiveness of Hib vaccine introduction in the national immunization program by province. The map indicates provinces where Hib vaccine in the national immunization program is cost-effective compared to the status quo when the ICER (US$/QALY gained) is less than the provincial GDP per capita and the Woods et al. threshold for China (US$ 4469). For Gansu province, the GDP per capita (US$ 4313) is less than the Woods et al. threshold, and including Hib vaccine in the national immunization program is cost-effective at both thresholds

At the provincial level, adding Hib vaccine to the NIP was cost-effective in 15 and 11 of the 31 provinces when compared to the provincial GDP per capita and Woods et al. threshold (US$ 4,469), respectively (Fig. 2), and it was cost-saving in Qinghai, Tibet, and Xinjiang provinces—all in the west region. The provinces where Hib vaccination was not cost-effective had lower disease burden and higher vaccine coverage in the private market compared to the other provinces. Conversely, the three provinces where Hib vaccine was cost-saving had high Hib incidence, high CFRs, and lower vaccine coverage in the private market compared to other provinces (Additional file 6: Table S2).

### Sensitivity analysis

**Fig. 3:**
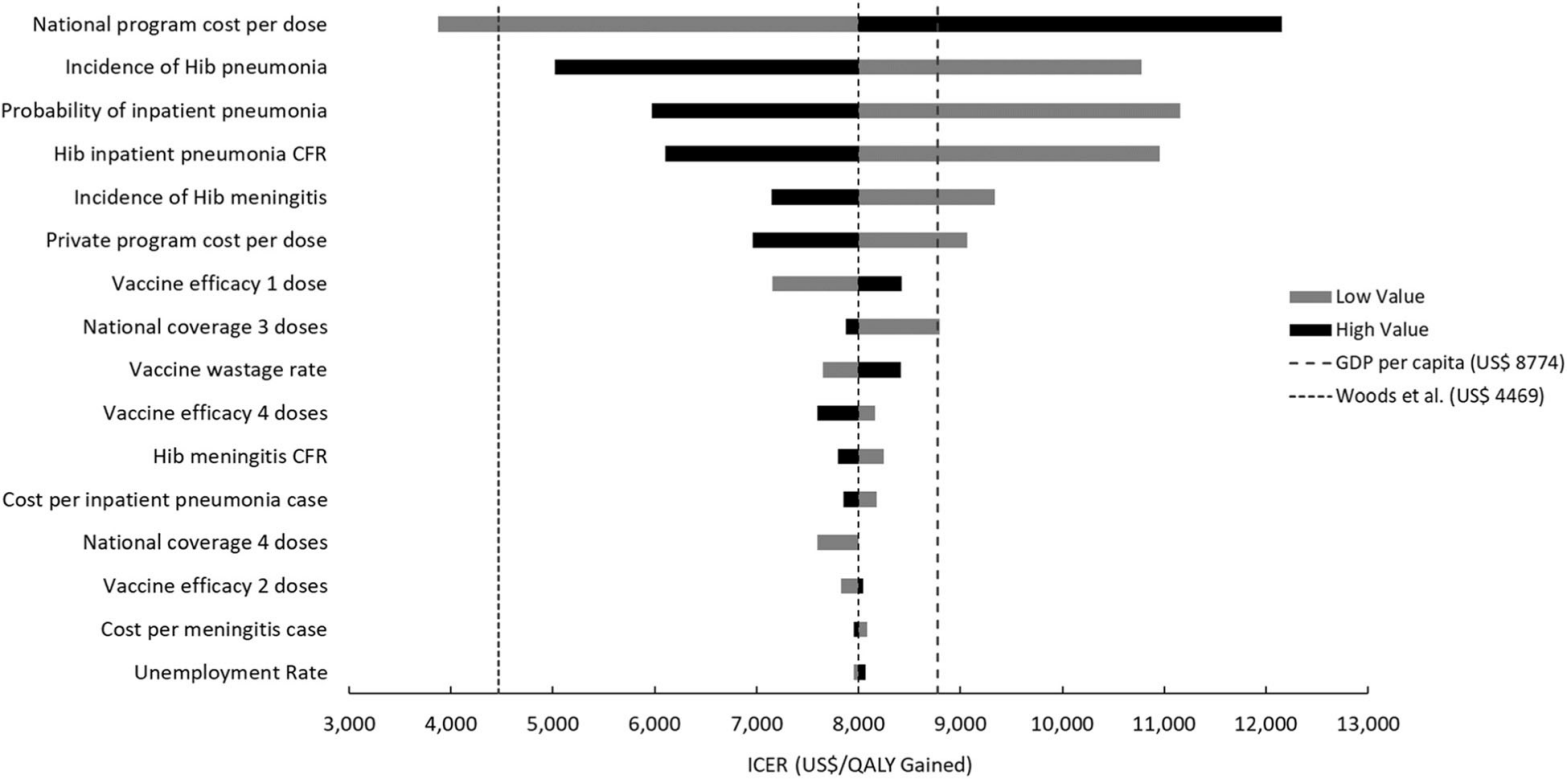
Tornado diagram of one-way sensitivity analyses for the most influential model parameters on ICER (US$/QALY gained)

**Fig. 4:**
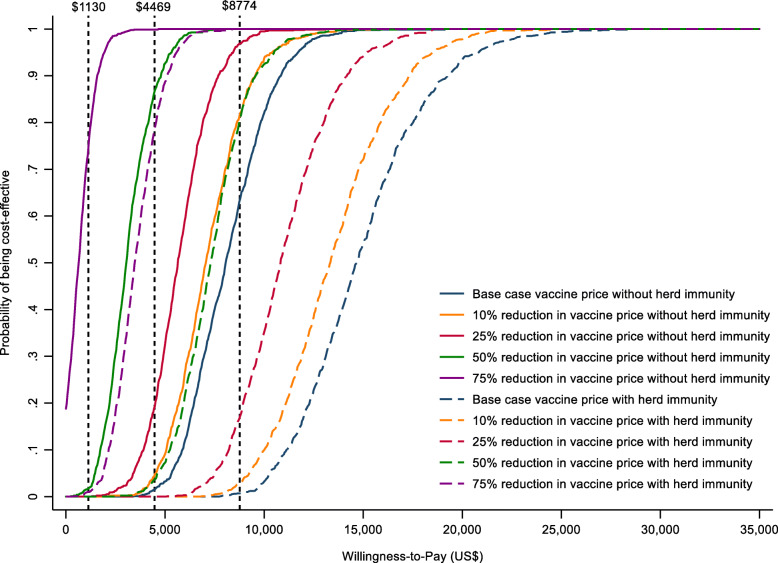
Cost-effectiveness acceptability curves of the national Hib vaccine program for the base case and different vaccine price and herd immunity scenarios. Vertical lines represent the national GDP per capita (US$ 8774), upper bound threshold (US$ 4469) by Woods et al. (2016), and lower bound threshold (US$ 1130) by Woods et al. (2016). The probability that adding Hib vaccine into the National Immunization Program is cost-effective for the base case and when reducing the price of Hib vaccine and accounting for the herd immunity from the probabilistic sensitivity analysis (PSA)

In deterministic sensitivity analyses, Hib vaccination remained cost-effective when varying the model parameters (Fig. 3). The most important parameters were the price per dose in the NIP and disease burden parameters for Hib pneumonia and meningitis, including incidence and CFR. In the PSA, Hib vaccine in the NIP had a 64% probability of being cost-effective nationally compared to the national GDP per capita, and the probability increased to more than 80% when reducing the price of Hib vaccine by at least 10% (Fig. 4).

Including herd immunity in the model decreased the cost-effectiveness nationally and in most provinces. Herd immunity did not have any effect on the NIP strategy because of the high direct vaccine coverage in all provinces. However, accounting for herd immunity increased the vaccine effective coverage for the *status quo* strategy where the average provincial coverage in the private market was 33%. More socioeconomically developed areas (e.g., Shanghai and Beijing) had higher vaccine coverage in the private market and greater herd immunity effects that decreased cost-effectiveness. Although the national ICER per QALY gained increased from US$ 8001 to US$ 14,903 when accounting for herd immunity, Hib vaccine in the NIP remained cost-effective in 9 provinces. It did not become cost-effective until the NIP vaccine price decreased by 50% or more. Nationally, adding Hib vaccine to the NIP became cost-saving when the price per dose was less than US$ 2.02. A 3-dose schedule also increased cost-effectiveness overall (US$ 4071 per QALY gained nationally) with Hib vaccine in the NIP becoming cost-effective in 20 of 31 provinces (Additional file 7: Table S1).

## Discussion

Currently, China is the only country not including Hib vaccine in its NIP, and continues to have the largest population of children without access to Hib vaccine despite availability in the private market. This is the first study at the provincial level to estimate the cost-effectiveness of introducing Hib vaccine into the NIP compared to the *status quo* in the private market. For a single birth cohort in 2017, the introduction of Hib vaccine into the NIP could save approximately 2700 lives and avert over 235,600 cases of Hib invasive disease. Averting premature death and long-term disability from Hib sequelae resulted in savings of $ 384 million in averted treatment costs and increased lifetime productivity from averted premature death. With an ICER per QALY gained of US$ 8001, Hib vaccine in the NIP was cost-effective nationally compared to the 2017 GDP per capita. Adding Hib vaccine to China’s NIP is not only cost-effective but would also expand access to the vaccine for children throughout China. Although this is the first study to compare national vaccination with the *status quo* in the private market, our findings at the national level are consistent with other studies comparing national vaccination to no vaccination [43, 44].

At the provincial level, Hib vaccine in the NIP was cost-effective in 15 of 31 provinces with provinces in the west region getting the largest benefit. Expanded Hib vaccination was most cost-effective in provinces with low coverage in the private market and/or higher Hib disease burden. While other programs and interventions aimed at improving maternal and child health had been implemented throughout China in the last decade [45, 46], none of these prevented Hib disease, and in less socioeconomically developed provinces, mostly in the west region, Hib disease burden remained high while access to vaccines in the private market was limited. In Qinghai, Tibet, and Xinjiang, all provinces with low Hib vaccine coverage in the private market and high mortality, introducing Hib vaccine into these provinces was not only cost-effective but also cost-saving. Introducing Hib vaccine into China’s NIP could not only effectively reduce the disease burden especially in the west region, but it would also promote health equity by improving vaccine access in less socioeconomically developed and higher disease burden provinces.

For the base case analysis, we used a conservative approach to estimate model parameters, but the sensitivity analyses demonstrated the robustness of the study results. Vaccine price, incidence, and CFR were among the main drivers of cost-effectiveness. In the absence of reliable vaccine price data in China, which was not Gavi eligible, we assumed the NIP vaccine price was the same as the private market. The Chinese government is likely to negotiate a reduced price for Hib vaccines when purchasing a very large volume per year if Hib vaccine is added to the NIP. Nationally, Hib vaccine, which was already cost-effective in the base case analysis, would become cost-saving if the vaccine price could drop from US$ 11.62 to US$ 2.02. Regionally, Hib vaccination became cost-effective in all three regions at US$ 5.82.

This study had some limitations. First, reliable data on access to care for each province and deaths occurring outside health facilities were unavailable. Nationally, access to care was high in China, and we assumed all Hib cases sought care at a health facility and all deaths occurred in hospitals. Reductions in care-seeking would reduce cost savings and increase ICER estimates. Conversely, deaths occurring outside health facilities could result in an underestimate of ICER values. Second, the model did not account for the dispersion of children vaccinated in the private market in the community. We assumed coverage was equally distributed throughout the province, but in reality, there are likely pockets of unvaccinated children and pockets with higher vaccine coverage where indirect effects could be present.

Third, the analysis relied on treatment cost data from CHIRA that has some limitations. While CHIRA cost data were nationally representative, it might overestimate the cost of Hib-related disease because data came from mostly urban areas and might not represent the rural populations. This urban bias might result in an underestimate of the ICERs in provinces with large rural populations and overestimate the cost-effectiveness. Despite this limitation, the sensitivity analysis showed that the vaccine price and disease burden parameters were the largest drivers of the ICER estimates, and varying in the cost of treatment was not likely to change the cost-effectiveness in individual provinces. Similarly, the cases included in the CHIRA database included both laboratory-confirmed and suspected cases that could bias the treatment cost estimates. However, in China, high antibiotic use makes it difficult to laboratory-confirm Hib cases, and most laboratories do not detect the majority of Hib disease. Due to the low confirmation rates in China, the treatment of confirmed and non-confirmed cases of suspected bacterial origin was similar, and non-confirmed cost data were not likely to significantly affect the overall treatment cost estimates.

Fourth, incidence and treatment costs of Hib sequelae were not available by province, so national estimates obtained from published literature were used in the model. The deterministic sensitivity analysis showed that the impact of Hib sequelae parameters was minimal compared to other parameters, but this could be an underestimate because the treatment cost estimates used did not account for lifelong costs to treat the sequelae.

## Conclusions

Despite these limitations, Hib vaccine was cost-effective at the national level, in several provinces in China, and even cost-saving in three west provinces. This study provides evidence to support the introduction of Hib vaccine into China’s NIP. Although Hib vaccine was available in the private market, the majority of children, especially those in poorer and higher burden areas, still lacked access because of the high price of Hib vaccines. The provincial analyses supported subnational introduction of Hib vaccine if no decision was made nationally, and priority should be given in provinces of Tibet, Xinjiang, and Qinghai due to their severe disease burden and substantial benefits gained from including Hib vaccine in their local immunization programs. Introduction of Hib vaccine in the NIP or in high burden provinces should be a key strategy to meet the Sustainable Development Goal child survival targets by 2030 and accelerate the elimination of Hib diseases globally.

## Supplementary Information

**Additional file 1: Table S1**- Model live birth cohort and mortality rate parameters by province

**Additional file 2: Table S2**- Province-specific disease burden model parameters and data sources; **Table S3**- Hib disease burden among children 1-59 months by province.

**Additional file 3: Table S4**- ICD-10 codes used for each syndrome; **Table S5**- Summary of individual-level cost data from 2013 to 2017 obtained from the China Healthcare Insurance Research Association (CHIRA); **Table S6**- Inputs for the calculations of indirect costs (US$); **Table S7**- Cost of illness and lifetime productivity estimates (US$).

**Additional file 4: Table S8**- Summary of Hib vaccine products available in China; **Table S9**- Provincial vaccine coverage and weighted vaccine coverage in the private in 2017; **Table S10**- Regional dose-specific and weighted combined Hib vaccine coverage used for the NIP strategy; **Table S11**- Societal cost per dose of the National Immunization Program (NIP) in China in 2015 (2017 US$).

**Additional file 5: Table S12**- Provincial disease burden and economic supplemental results; **Table S13**- Provincial discounted economic costs of Hib disease and vaccine program costs (2017 US$) for each vaccination strategy from the societal perspective.

**Additional file 6: Table S14**- Incremental cost-effectiveness ratios of including Hib vaccine in the NIP in 2017 (RMB); **Table S15**- Incremental cost-effectiveness ratios of including Hib vaccine in the NIP in 2017 (US$); **Table S16**- Hib cases and deaths averted by province and region when including herd immunity effects.

**Additional file 7: Table S17**- Incremental cost-effectiveness ratios (US$) for the different Hib vaccine schedules (4 doses vs. 3 doses in the NIP); **Table S18**- National incremental cost-effectiveness ratios (US$ per QALY gained) when varying the NIP Hib vaccine price; **Table S19**- Provincial incremental cost-effectiveness ratios (US$ per QALY gained) when reducing the NIP Hib vaccine price.

## Data Availability

The datasets generated and analyzed during the current study are available from the corresponding author (Hai Fang) on reasonable request.

## Abbreviations

Hib: Haemophilus influenzae type b
NIP: National immunization program
CDC: Center for disease control and prevention
WHO: World health organization
ICER: Incremental cost-effectiveness ratios
QALY: Quality-adjusted life year
NPNM: Non-pneumonia non-meningitis
CHIRA: China healthcare insurance research association
GDP: Gross domestic product
DSA: Deterministic sensitivity analyses
PSA: Probabilistic sensitivity analysis
