# Supplementary material for: National and provincial impact and cost-effectiveness of Haemophilus influenzae type b conjugate vaccine in China: a modeling analysis
Source: BMC Med. 2021 Aug 11;19:181. doi: 10.1186/s12916-021-02049-7 (PMC8356460; doi:10.1186/s12916-021-02049-7)
Supplement: Supplementary file 1 — Additional file 1: Table S1- Model live birth cohort and mortality rate parameters by province [file 12916_2021_2049_MOESM1_ESM.docx]

**Additional file 1. Data sources for demographic model parameters in economic model**

Province-level live birth cohort data for 2017 were obtained from the 2018 China Health and Family Planning Statistical Yearbook.[1] All-cause mortality rates for neonates and under-five year-olds were obtained from a 2016 cause of death systematic review[2] and the 2017 Global Burden of Disease (GBD) study in China, respectively.[3] Song et al. 2016 identified high-quality community-based longitudinal cause of death studies in under-five children in China and modeled all-cause mortality by province for 2015.[2] For the economic model, we assumed the mortality rates remained stable from 2015 to 2017. The provincial birth cohorts and mortality rates used in the economic model are described in Table 1.

**Table 1. Model live birth cohort and mortality rate parameters by province**

| **Province** | **GDP Per Capita in RMB (US$)** | **2017 Live Birth Cohort** | **Neonatal mortality rate**  **(per 1,000 live births)** | **Under five mortality rate**  **(per 1,000 live births)** |
| --- | --- | --- | --- | --- |
| Anhui | 44,206 (6,501) | 773,521 | 5.5 | 12.0 |
| Beijing | 128,927 (18,960) | 242,812 | 1.9 | 5.0 |
| Chongqing | 63,689 (9,366) | 317,689 | 6.0 | 13.0 |
| Fujian | 82,976 (12,202) | 615,152 | 4.0 | 7.0 |
| Gansu | 29,326 (4,313) | 357,070 | 10.8 | 20.0 |
| Guangdong | 81,089 (11,925) | 1,952,085 | 2.7 | 7.0 |
| Guangxi | 41,955 (6,170) | 819,427 | 5.3 | 12.0 |
| Guizhou | 37,956 (5,582) | 617,176 | 9.0 | 18.0 |
| Hainan | 48,430 (7,122) | 134,657 | 7.5 | 16.0 |
| Hebei | 47,985 (7,057) | 961,194 | 5.9 | 13.0 |
| Heilongjiang | 42,699 (6,279) | 177,658 | 5.4 | 12.0 |
| Henan | 47,130 (6,931) | 1,420,062 | 5.7 | 13.0 |
| Hubei | 61,972 (9,114) | 652,625 | 5.3 | 12.0 |
| Hunan | 50,563 (7,436) | 804,752 | 4.7 | 9.0 |
| Inner Mongolia | 63,786 (9,380) | 211,577 | 6.6 | 13.0 |
| Jiangsu | 107,189 (15,763) | 806,974 | 2.5 | 7.0 |
| Jiangxi | 45,187 (6,645) | 606,831 | 7.3 | 16.0 |
| Jilin | 56,102 (8,250) | 168,275 | 3.5 | 9.0 |
| Liaoning | 54,745 (8,051) | 307,602 | 3.6 | 7.0 |
| Ningxia | 50,917 (7,488) | 104,561 | 7.9 | 16.0 |
| Qinghai | 44,348 (6,522) | 77,215 | 9.2 | 21.0 |
| Shaanxi | 57,266 (8,421) | 470,979 | 6.9 | 15.0 |
| Shandong | 72,851 (10,713) | 1,655,895 | 4.2 | 10.0 |
| Shanghai | 124,571 (18,319) | 199,072 | 2.8 | 7.0 |
| Shanxi | 40,557 (5,964) | 379,105 | 4.4 | 12.0 |
| Sichuan | 44,651 (6,566) | 863,843 | 6.6 | 15.0 |
| Tianjin | 119,238 (17,535) | 121,771 | 3.2 | 8.0 |
| Tibet | 39,259 (5,773) | 50,000 | 16.5 | 36.0 |
| Xinjiang | 45,099 (6,632) | 313,782 | 13.2 | 28.0 |
| Yunnan | 34,545 (5,080) | 684,617 | 8.4 | 17.0 |
| Zhejiang | 92,057 (13,538) | 710,836 | 3.3 | 7.0 |

**REFERENCES**

1. National Health and Family Planning Commission of China: China Health

and Family Planning Statistical Yearbook 2018. Chinese Academy of Medical

Sciences & Peking Union Medical College Press. Beijing, 2018.

2. Song P, Theodoratou E, Li X, Liu L, Chu Y, Black RE, Campbell H, Rudan I, Chan KY. Causes of death in children younger than five years in China in 2015: an updated analysis. J Glob Health.2016; 6(2):020802.

3. Zhou M, Wang H, Zeng X, Yin P, Zhu J, Chen W, Li X, Wang L, Wang L, Liu Y et al. Mortality, morbidity, and risk factors in China and its provinces, 1990-2017: a systematic analysis for the Global Burden of Disease Study 2017. Lancet.2019; 394(10204):1145-1158.
