# Supplementary material for: National and provincial impact and cost-effectiveness of Haemophilus influenzae type b conjugate vaccine in China: a modeling analysis
Source: BMC Med. 2021 Aug 11;19:181. doi: 10.1186/s12916-021-02049-7 (PMC8356460; doi:10.1186/s12916-021-02049-7)
Supplement: Supplementary file 2 — Additional file 2: Table S2- Province-specific disease burden model parameters and data sources; Table S3- Hib disease burden among children 1-59 months by province. [file 12916_2021_2049_MOESM2_ESM.docx]

**Additional file 2.** **Data sources and methods for estimating Hib epidemiological parameters by province**

We estimated the Hib disease burden in children aged 1-59 months separately for the three clinical syndromes associated with Hib: pneumonia, meningitis, and invasive non-pneumonia, non-meningitis disease (NPNM). The estimated, syndrome-specific incidence from the disease burden model was used for this economic analysis [1]. The case definitions used methods previously described [2]. Here, we briefly describe the methods used in the disease burden model and the data sources and all key parameters are presented in Table 1.

To estimate the burden of Hib pneumonia, we applied estimates of the proportion of pneumonia deaths and cases attributable to Hib to annual, provincial, all-cause pneumonia mortality and morbidity estimates obtained from the GBD study [3]. The Hib attributable fraction for pneumonia mortality was estimated using efficacy against radiograph-confirmed, primary endpoint pneumonia from vaccine clinical trials.[2] Efficacy against clinical severe pneumonia was used to estimate clinical severe Hib pneumonia morbidity. Provincial Hib pneumonia CFR values were estimated by dividing Hib pneumonia mortality by clinical severe Hib pneumonia cases (See Table 2).

For meningitis, we used estimates of the proportion of meningitis cases attributable to common bacterial pathogens and estimates of relative case fatality for these pathogens to estimate the proportion of Hib meningitis deaths also as described in a previous publication.[2] For each province, we prepared summary estimates of each parameter by meta-analyzing data reported in previous literatures from Asia and other epidemiologically relevant settings. Pathogen-specific meningitis deaths were estimated by applying the proportion of meningitis deaths caused by Hib to modeled provincial all-cause deaths of meningitis aged 1-59 months prepared by the GBD study in China.

Hib morbidity from NPNM was estimated by using the meta-estimated ratio of Hib NPNM to Hib meningitis obtained from published studies globally, stratified by all-cause child mortality.[2] Similarly, we estimated NPNM deaths by multiplying NPNM severe cases by the meningitis CFR and the NPNM-to-meningitis CFR ratio obtained from stratified meta-estimates of the published literature. Hib morbidity and mortality estimates were prepared assuming no vaccine use. We then adjusted these estimates to account for provincial dose-specific vaccine effective coverage in the economic model.[4]

Global Hib meningitis and Hib NPNM CRF values stratified by all-cause child mortality strata were used at the provincial level. All provinces except Tibet and Xinjiang were in the same all-cause child mortality stratum, and the same CRF values for Hib meningitis (0.044; UR 0.033-0.055) and Hib NPNM (0.001; UR 0.0008-0.0013) were applied. The CRF values for Hib meningitis and Hib NPNM for Tibet were 0.216 (UR 0.0636-0.7328) and 0.005 (UR 0.0015-0.0173), respectively. The CRF values for Hib meningitis and Hib NPNM for Xinjiang were 0.120 (UR 0.0313-0.4603) and 0.003 (SUR 0.0007-0.0109), respectively.

| **Table 1. Province-specific disease burden model parameters and data sources** | | |
| --- | --- | --- |
| **Model Parameter** | **National estimates (UR)** | **Source of data** |
| **Pathogen-specific pneumonia (lower respiratory infection)** |  |  |
| All-cause pneumonia deaths | 19614 (16891-22794) | Modelled estimates based on the Global Disease Burden (GBD) study from China^[3]^ |
| All-cause pneumonia cases | 3473515 (2678883-4419332) | Modelled estimates based on the Global Disease Burden (GBD) study from China^[3]^ |
| Proportion of pneumonia deaths attributable to Hib | 21.3% (0.4%-42.1%) | Global estimates with clinical trial data^[2]^ |
| Proportion of pneumonia cases attributable to Hib | 5% (1.1%-8.8%) | Global estimates with clinical trial data^[2]^ |
| **Pathogen-specific meningitis** |  |  |
| All-cause meningitis deaths | 1945 (1623-2302) | Modelled estimates based on the Global Disease Burden (GBD) study from China^[3]^ |
| Proportion of meningitis cases attributable to Hib | 45.2% (39.3%-51.1%) | Meningitis surveillance from litertures |
| Hib meningitis CFR | See Additional file 1 | Hib meningitis surveillance from litertures |
| **Pathogen-specific NPNM** |  |  |
| Hib NPNM case multiplier | 0.337 (0.038-0.636) | Hib disease surveillance from from litertures |
| Hib NPNM CFR multiplier | 0.024 (-0.082-0.129) | Hib disease surveillance from from litertures |
| **Population at risk and demographic model parameters** |  |  |
| Child mortality | See Additional file 1 | Modelled estimates based on the Global Disease Burden (GBD) study from China^[3]^ |
| Child population | See Additional file 1 | China National Bureau of Statistics in 2017， Child Immunization Population Statistics in 2017 from China CDC |
| Hib vaccine doses | 17549473 | China CDC in 2017 |

[2][4]

The incidence estimates used in the economic model assumed no vaccine effect (i.e., no Hib vaccination publicly or privately) because the vaccine effect was applied in the Markov model based on the vaccination strategy and corresponding coverage and costs. Because access to care was high in all provinces,[5] we assumed all severe diseases (severe pneumonia, meningitis, and NPNM) resulted in hospitalization, and all deaths occurred in hospitals.

[5]

**Table 2. Hib disease burden among children 1-59 months by province**

| **Province** | **Hib pneumonia incidence per 100,000 (UR)** | **Hib meningitis incidence per 100,000 (UR)** | **Hib NPNM incidence per 100,000 (UR)** | **Inpatient Hib pneumonia CFR**  **(UR)** |
| --- | --- | --- | --- | --- |
| Anhui | 393 (358-641) | 7 (4-13) | 3 (1-5) | 0.045 (0.016-0.099) |
| Beijing | 552 (504-902) | 4 (2-8) | 1 (1-3) | 0.023 (0.008-0.050) |
| Chongqing | 468 (427-764) | 6 (3-11) | 2 (1-4) | 0.037 (0.013-0.080) |
| Fujian | 389 (356-636) | 5 (2-10) | 2 (1-3) | 0.038 (0.013-0.082) |
| Gansu | 482 (440-788) | 23 (11-41) | 8 (4-14) | 0.101 (0.035-0.222) |
| Guangdong | 580 (529-947) | 6 (3-12) | 2 (1-4) | 0.031 (0.011-0.068) |
| Guangxi | 389 (355-636) | 6 (3-10) | 2 (1-4) | 0.049 (0.017-0.107) |
| Guizhou | 364 (333-595) | 5 (2-9) | 2 (1-3) | 0.058 (0.020-0.127) |
| Hainan | 436 (398-713) | 13 (6-24) | 4 (2-8) | 0.122 (0.042-0.267) |
| Hebei | 433 (396-708) | 16 (7-29) | 5 (3-10) | 0.056 (0.020-0.123) |
| Heilongjiang | 545 (497-890) | 13 (6-23) | 4 (2-8) | 0.036 (0.013-0.079) |
| Henan | 322 (294-526) | 8 (4-15) | 3 (1-5) | 0.044 (0.015-0.097) |
| Hubei | 370 (337-604) | 4 (2-7) | 1 (1-2) | 0.035 (0.012-0.077) |
| Hunan | 338 (308-552) | 3 (1-5) | 1 (0-2) | 0.027 (0.009-0.059) |
| Inner Mongolia | 541 (494-883) | 18 (8-33) | 6 (3-11) | 0.062 (0.022-0.137) |
| Jiangsu | 404 (369-661) | 3 (1-5) | 1 (0-2) | 0.013 (0.005-0.029) |
| Jiangxi | 396 (362-648) | 15 (7-27) | 5 (2-9) | 0.107 (0.037-0.236) |
| Jilin | 595 (543-973) | 17 (8-30) | 6 (3-10) | 0.042 (0.015-0.093) |
| Liaoning | 571 (522-933) | 4 (2-8) | 1 (1-3) | 0.013 (0.005-0.029) |
| Ningxia | 446 (407-729) | 15 (7-26) | 5 (2-9) | 0.102 (0.036-0.225) |
| Qinghai | 510 (465-833) | 30 (14-55) | 10 (5-19) | 0.179 (0.063-0.394) |
| Shaanxi | 427 (390-697) | 18 (8-32) | 6 (3-11) | 0.094 (0.033-0.206) |
| Shandong | 334 (305-546) | 4 (2-7) | 1 (1-2) | 0.016 (0.006-0.035) |
| Shanghai | 566 (516-924) | 5 (2-8) | 2 (1-3) | 0.024 (0.008-0.052) |
| Shanxi | 452 (412-738) | 17 (8-31) | 6 (3-11) | 0.080 (0.028-0.176) |
| Sichuan | 563 (514-920) | 10 (5-18) | 3 (2-6) | 0.072 (0.025-0.159) |
| Tianjin | 542 (495-885) | 7 (3-13) | 2 (1-4) | 0.039 (0.014-0.086) |
| Tibet | 649 (593-1060) | 12 (5-18) | 4 (2-6) | 0.254 (0.088-0.557) |
| Xinjiang | 455 (416-744) | 14 (5-18) | 5 (2-6) | 0.244 (0.085-0.535) |
| Yunnan | 634 (579-1035) | 17 (8-30) | 6 (3-10) | 0.126 (0.044-0.277) |
| Zhejiang | 550 (502-898) | 5 (2-9) | 2 (1-3) | 0.020 (0.007-0.044) |

UR Uncertainty range

**REFERENCES**

1. Xiaozhen Lai BW, Wenzhou Yu, Tingting Xu, Haijun Zhang, Cristina Garcia,Yan Guo, Zundong Yin, Maria Deloria Knoll, Hai Fang: National, regional, and provincial disease burden associated with *Streptococcus pneumoniae* and *Haemophilus influenzae* type b in children in China: modelled estimates for 2010–17. In*.*; 2021.

2. Wahl B, O'Brien KL, Greenbaum A, Majumder A, Liu L, Chu Y, Lukšić I, Nair H, McAllister DA, Campbell H *et al*. Burden of Streptococcus pneumoniae and Haemophilus influenzae type b disease in children in the era of conjugate vaccines: global, regional, and national estimates for 2000-15. *Lancet Glob Health.*2018; 6(7):e744-e757.

3. Zhou M, Wang H, Zeng X, Yin P, Zhu J, Chen W, Li X, Wang L, Wang L, Liu Y *et al*. Mortality, morbidity, and risk factors in China and its provinces, 1990-2017: a systematic analysis for the Global Burden of Disease Study 2017. *Lancet (London, England).*2019; 394(10204):1145-1158.

4. Watt JP, Wolfson LJ, O'Brien KL, Henkle E, Deloria-Knoll M, McCall N, Lee E, Levine OS, Hajjeh R, Mulholland K *et al*. Burden of disease caused by Haemophilus influenzae type b in children younger than 5 years: global estimates. *Lancet.*2009; 374(9693):903–911.

5. He C, Liu L, Chu Y, Perin J, Dai L, Li X, Miao L, Kang L, Li Q, Scherpbier R *et al*. National and subnational all-cause and cause-specific child mortality in China, 1996-2015: a systematic analysis with implications for the Sustainable Development Goals. *Lancet Glob Health.*2017; 5(2):e186-e197.
