# Supplementary material for: National and provincial impact and cost-effectiveness of Haemophilus influenzae type b conjugate vaccine in China: a modeling analysis
Source: BMC Med. 2021 Aug 11;19:181. doi: 10.1186/s12916-021-02049-7 (PMC8356460; doi:10.1186/s12916-021-02049-7)
Supplement: Supplementary file 3 — Additional file 3: Table S4- ICD-10 codes used for each syndrome; Table S5- Summary of individual-level cost data from 2013 to 2017 obtained from the China Healthcare Insurance Research Association (CHIRA); Table S6- Inputs for the calculations of indirect costs (US$); Table S7- Cost of illness and lifetime productivity estimates (US$). [file 12916_2021_2049_MOESM3_ESM.docx]

**Additional file 3.** **Methods for estimating direct and indirect costs of Hib diseases by province**

Direct medical costs of inpatient and outpatient pneumonia, meningitis, NPNM, and sequelae were estimated using individual-level health insurance data obtained from the China Healthcare Insurance Research Association (CHIRA). In 2008, the Chinese government established a routine reporting system for health service utilization of patients with Urban Basic Medical Insurance, which included available data from three sample cities in each province or municipalities in all 31 provinces of Mainland China. Hong Kong and Macao special administrative regions and Taiwan were excluded from this analysis. We extracted cost data from 2013–2017 from the CHIRA database. The data contained basic demographic information and medical service utility information (e.g., disease diagnosis, medical expenses, and days of hospitalization). CHIRA data were categorized by ICD-10 code, and the ICD-10 case definitions used for this analysis are described in Table 1.

**Table 1. ICD-10 codes used for each syndrome**

| **Disease Name** | **ICD-10 Codes** |
| --- | --- |
| Pneumonia | B01.2, J09-J18, J20- J21.0, P23.0- P23.9, |
| Meningitis | A17.0- A17.1, A20.3, A32.1, A39.0, A87, B00.3, B01.0, B02.1, B26.1, B37.5, B38.4, C70, D32, D42, G0, G00, G01, G02, G96.1 |
| NPNM | A02.1, A22.7, A26.7, A32.7, A40.0- A40.3, A41.0- A41.9 |
| Hemiplegia | G81.0, G81.1, G81.9 |
| Hearing Loss | H90- H91 |
| Cognitive Disability | F70-F79, F84.4, Z81.0 |
| Epilepsy | F80.3, G40, T42, Z82.0, X41, X61, Y11 |

Average inpatient and outpatient direct medical costs for pneumonia were estimated by province. Meningitis and NPNM direct medical costs were estimated by region due to their small sample sizes (See Table 2). Regional average cost per case estimates were weighted by the expected distribution of cases in each province. The direct medical cost of sequelae was estimated at the national level, and the cost of hearing loss sequelae included the average cost of cochlear implant multiplied by the probability of cochlear implant obtained from published literature. [1, 2] All CHIRA data were analyzed using Stata 15 (College Station, TX, USA).

**Table 2. Summary of individual-level cost data from 2013 – 2017 obtained from the China Healthcare Insurance Research Association (CHIRA)**

| **Disease** | **Sample size** | **Level of Cost Estimates Generated** |
| --- | --- | --- |
| Inpatient Pneumonia | 125,388 | Provincial |
| Outpatient Pneumonia | 55,374 | Provincial |
| Meningitis | 259 | Regional |
| NPNM | 3,177 | Regional |
| Hemiplegia | 69 | National |
| Hearing Loss | 221 | National |
| Cognitive Disability | 532 | National |
| Epilepsy | 2,493 | National |

Direct non-medical costs of inpatient pneumonia were estimated using data from surveys conducted by the China CDC from 2015 to 2016 in Gansu province.[3] A total of 4,006 children were surveyed, of which 76.4% were from urban areas. Direct non-medical costs included the cost of transportation, accommodation and other fees. The total direct non-medical cost for inpatient pneumonia in Gansu province was US$ 280 (￥1,903).[3] Direct non-medical costs of inpatient meningitis were estimated using the national average (US$ 1,554; equivalent to ￥10,567) from another survey conducted in 2014 by the China CDC in Hubei, Shandong and Hebei provinces.[4] Because data on the non-medical cost of NPNM were not available from the surveys by China CDC, the non-medical cost of inpatient NPNM was estimated by adjusting the non-medical cost of pneumonia by the ratio of inpatient hospitalization days between NPNM and pneumonia (6.6 days for pneumonia vs. 7.2 days for NPNM). To generate estimates for all provinces, all non-medical cost estimates were adjusted using the ratio of the provincial total household consumption expenditure in 2017 obtained from the China Statistics Yearbook. We assumed direct non-medical costs of outpatient pneumonia and NPNM only contained the cost of transportation and were equal to the transportation cost of vaccination from the surveys by China CDC.[5] See Table 3 for the assumptions used to estimate the direct and indirect costs for each province.

Indirect costs associated with caregiver productivity loss and future lifetime productivity loss due to premature death and disability was estimated using the human capital approach. Caregiver productivity loss was estimated using the average wage per day of employed persons in urban private units, average days of hospitalization, and average number of caregivers The average hospitalized days (lengths of stay) was estimated from the CHIRA database (See Table 3), and the average number of caregivers for pneumonia, meningitis, and NPNM were assumed to be 1.9, 2, and 2 caregivers, respectively, based on data from the surveys by China CDC.[3, 4] The indirect cost of outpatient pneumonia and NPNM were assumed to equal one half days’ productivity loss for caregivers.

Future costs after the first five years of life included the lifetime productivity loss due to disability and premature death and for cases with meningitis sequelae, the cost of special education. Lifetime productivity loss due to premature death was estimated using the national average annual wage rate for the average productive life years (16 to 60 years) multiplied by the unemployment rate and discounted to the year of vaccination. To estimate the lifetime productivity loss by province, the national lifetime productivity loss was adjusted using the ratio of annual salary in 2017 in each province. For cases with long-term sequelae from meningitis, we assumed the sequelae remained throughout individual's lifetime and reduced lifetime productivity compared to healthy individuals. The discounted average annual wage rate for the remaining productive life years was multiplied by the sequelae utility to estimate the lifetime productivity loss due to disability among cases with sequalae. We assumed special education was required for all sequelae included in the model. The annual cost of special education, derived from 2018 China Education Statistics Yearbook and China Education Expenditure Statistical Yearbook,[6, 7] was multiplied by the average years of special education (12 years). The provincial cost of illness and lifetime productivity estimates are presented in Table 4.

**Table 3. Inputs for the calculations of indirect costs (US $)**

| **Province** | **Average Annual Wage** | **Discounted Cost of Special Education (Age 6-18)** | **Discounted Lifetime Productivity** | **Unemployment Rate*** | **Household Consumption** | **Pneumonia** | | **Meningitis** | | **NPNM** | |
| --- | --- | --- | --- | --- | --- | --- | --- | --- | --- | --- | --- |
|  |  |  |  |  |  | **Days of Hospitalization** | **Productivity Loss of Caregivers** | **Days of Hospitalization** | **Productivity Loss of caregivers** | **Days of Hospitalization** | **Productivity Loss of Caregivers** |
| Anhui | 6058.7 | 59683.4 | 90832.2 | 2.9% | 2316.4 | 7.7 | 340.5 | 16.0 | 743.8 | 7.4 | 172.5 |
| Beijing | 10402.6 | 211226.9 | 155957.4 | 1.4% | 5503.7 | 7.3 | 549.8 | 19.1 | 1525.7 | 8.5 | 338.6 |
| Chongqing | 7419.1 | 75670.7 | 111228.1 | 3.4% | 2632.1 | 6.6 | 358.6 | 17.5 | 997.3 | 10.1 | 287.0 |
| Fujian | 7180.9 | 95771.7 | 107656.4 | 3.9% | 3124.9 | 7.1 | 372.7 | 19.1 | 1053.2 | 8.5 | 233.7 |
| Gansu | 5544.7 | 66037.6 | 83126.7 | 2.7% | 1929.4 | 7.4 | 297.9 | 17.5 | 745.3 | 10.1 | 214.5 |
| Guangdong | 7845.1 | 129829.9 | 117615.1 | 2.5% | 3649.9 | 7.3 | 417.5 | 19.1 | 1150.6 | 8.5 | 255.3 |
| Guangxi | 5621.6 | 50283.3 | 84279.8 | 2.2% | 1974.1 | 7.2 | 293.4 | 17.5 | 755.7 | 10.1 | 217.4 |
| Guizhou | 6146.5 | 41925.9 | 92148.4 | 3.2% | 1907.3 | 11.6 | 520.4 | 17.5 | 826.2 | 10.1 | 237.7 |
| Hainan | 6711.8 | 99395.3 | 100623.4 | 2.3% | 2265.1 | 6.7 | 325.9 | 16.0 | 824.0 | 7.4 | 191.1 |
| Hebei | 5608.2 | 65057.2 | 84079.2 | 3.7% | 2270.1 | 8.1 | 329.1 | 16.0 | 688.5 | 7.4 | 159.7 |
| Heilongjiang | 4767.9 | 68997.2 | 71481.4 | 4.2% | 2290.8 | 9.4 | 324.9 | 16.0 | 585.4 | 7.4 | 135.8 |
| Henan | 5401.5 | 56360.8 | 80979.3 | 2.8% | 2019.1 | 7.8 | 304.7 | 16.0 | 663.2 | 7.4 | 153.8 |
| Hubei | 5462.1 | 80857.9 | 81887.7 | 2.6% | 2490.8 | 7.5 | 296.6 | 16.0 | 670.6 | 7.4 | 155.5 |
| Hunan | 5437.9 | 70516.8 | 81526.1 | 4.0% | 2523.6 | 5.7 | 224.1 | 16.0 | 667.6 | 7.4 | 154.8 |
| Inner Mongolia | 5386.2 | 92107.8 | 80750.0 | 3.6% | 2786.1 | 8.0 | 314.1 | 17.5 | 724.0 | 10.1 | 208.3 |
| Jiangsu | 7256.6 | 102229.7 | 108791.8 | 3.0% | 3451.3 | 7.9 | 418.9 | 19.1 | 1064.3 | 8.5 | 236.2 |
| Jiangxi | 5927.9 | 61496.6 | 88872.2 | 3.3% | 2126.3 | 7.0 | 301.6 | 16.0 | 727.8 | 7.4 | 168.8 |
| Jilin | 4883.7 | 94074.7 | 73216.5 | 3.5% | 2298.8 | 9.2 | 327.1 | 16.0 | 599.6 | 7.4 | 139.0 |
| Liaoning | 5243.2 | 93910.4 | 78607.0 | 3.8% | 3009.3 | 8.6 | 327.1 | 19.1 | 769.0 | 8.5 | 170.7 |
| Ningxia | 5732.6 | 78301.6 | 85944.3 | 3.9% | 2257.4 | 7.6 | 315.5 | 17.5 | 770.6 | 10.1 | 221.7 |
| Qinghai | 5380.6 | 86660.2 | 80666.3 | 3.1% | 2279.9 | 9.8 | 383.5 | 17.5 | 723.3 | 10.1 | 208.1 |
| Shaanxi | 5510.6 | 87880.8 | 82615.2 | 3.3% | 2191.1 | 7.1 | 285.6 | 17.5 | 740.8 | 10.1 | 213.1 |
| Shandong | 7645.9 | 91895.8 | 114627.7 | 3.4% | 2541.3 | 7.6 | 425.2 | 19.1 | 1121.4 | 8.5 | 248.9 |
| Shanghai | 7652.6 | 186054.8 | 114729.2 | 3.9% | 5851.8 | 7.3 | 404.4 | 19.1 | 1122.4 | 8.5 | 249.1 |
| Shanxi | 4668.4 | 72098.6 | 69988.8 | 3.4% | 2009.5 | 9.0 | 304.8 | 16.0 | 573.1 | 7.4 | 132.9 |
| Sichuan | 5895.1 | 58288.7 | 88380.6 | 4.0% | 2379.4 | 7.1 | 303.0 | 17.5 | 792.5 | 10.1 | 228.0 |
| Tianjin | 8785.3 | 120153.3 | 131709.9 | 3.5% | 4094.3 | 7.0 | 445.8 | 19.1 | 1288.5 | 8.5 | 285.9 |
| Tibet | 5876.2 | 138039.5 | 88096.2 | 2.7% | 1517.7 | 9.1 | 390.6 | 17.5 | 789.9 | 10.1 | 227.3 |
| Xinjiang | 5876.2 | 100495.4 | 88096.2 | 2.6% | 2218.7 | 6.5 | 278.5 | 17.5 | 789.9 | 10.1 | 227.3 |
| Yunnan | 5978.8 | 68622.7 | 89635.0 | 3.2% | 1861.5 | 8.1 | 353.0 | 17.5 | 803.7 | 10.1 | 231.3 |
| Zhejiang | 7101.3 | 110418.3 | 106463.7 | 2.7% | 3982.2 | 7.3 | 375.8 | 19.1 | 1041.5 | 8.5 | 231.1 |

*The unemployment rate was assumed to have a triangular distribution and range of 1.0% - 5.0% for deterministic and probabilistic sensitivity analysis.

**Table 4. Cost of illness and lifetime productivity estimates (US$)**

| **Province** | **Cost per inpatient pneumonia case**  **(Standard Deviation)** | **Cost per outpatient pneumonia case (Standard Deviation)** | **Cost per inpatient meningitis case (Standard Deviation)** | **Cost per inpatient NPNM case**  **(Standard Deviation)** | **Discounted Cost of special education* (Standard Deviation)** | **Discounted Lifetime productivity per capita (Standard Deviation)** |
| --- | --- | --- | --- | --- | --- | --- |
| Anhui | 1286 (161) | 32 (4) | 4405 (551) | 1791 (224) | 59683 (7460) | 90832 (11354) |
| Beijing | 2115 (264) | 66 (8) | 8509 (1064) | 2430 (304) | 211227 (26403) | 155957 (19495) |
| Chongqing | 1245 (156) | 52 (7) | 4875 (609) | 2418 (302) | 75671 (9459) | 111228 (13904) |
| Fujian | 1372 (172) | 46 (6) | 6661 (833) | 1975 (247) | 95772 (11971) | 107656 (13457) |
| Gansu | 1128 (141) | 108 (13) | 4215 (527) | 2241 (280) | 66038 (8255) | 83127 (10391) |
| Guangdong | 1635 (204) | 47 (6) | 7062 (883) | 2074 (259) | 129830 (16229) | 117615 (14702) |
| Guangxi | 1057 (132) | 33 (4) | 4251 (531) | 2251 (281) | 50283 (6285) | 84280 (10535) |
| Guizhou | 1757 (220) | 110 (14) | 4284 (536) | 2262 (283) | 41926 (5241) | 92148 (11519) |
| Hainan | 1403 (175) | 35 (4) | 4457 (557) | 1803 (225) | 99395 (12424) | 100623 (12578) |
| Hebei | 1413 (177) | 30 (4) | 4323 (540) | 1770 (221) | 65057 (8132) | 84079 (10510) |
| Heilongjiang | 1536 (192) | 41 (5) | 4230 (529) | 1748 (219) | 68997 (8625) | 71481 (8935) |
| Henan | 1242 (155) | 30 (4) | 4152 (519) | 1728 (216) | 56361 (7045) | 80979 (10122) |
| Hubei | 1244 (156) | 38 (5) | 4432 (554) | 1798 (225) | 80858 (10107) | 81888 (10236) |
| Hunan | 1144 (143) | 30 (4) | 4448 (556) | 1802 (225) | 70517 (8815) | 81526 (10191) |
| Inner Mongolia | 1484 (186) | 108 (13) | 4688 (586) | 2359 (295) | 92108 (11513) | 80750 (10094) |
| Jiangsu | 1645 (206) | 53 (7) | 6860 (858) | 2025 (253) | 102230 (12779) | 108792 (13599) |
| Jiangxi | 1108 (138) | 27 (3) | 4279 (535) | 1759 (220) | 61497 (7687) | 88872 (11109) |
| Jilin | 1346 (168) | 38 (5) | 4249 (531) | 1753 (219) | 94075 (11759) | 73217 (9152) |
| Liaoning | 1577 (197) | 42 (5) | 6307 (788) | 1893 (237) | 93910 (11739) | 78607 (9826) |
| Ningxia | 1109 (139) | 30 (4) | 4430 (554) | 2297 (287) | 78302 (9788) | 85944 (10743) |
| Qinghai | 1486 (186) | 108 (13) | 4395 (549) | 2286 (286) | 86660 (10833) | 80666 (10083) |
| Shaanxi | 1190 (149) | 108 (14) | 4361 (545) | 2278 (285) | 87881 (10985) | 82615 (10327) |
| Shandong | 1327 (166) | 52 (6) | 6393 (799) | 1907 (238) | 91896 (11487) | 114628 (14328) |
| Shanghai | 2017 (252) | 56 (7) | 8303 (1038) | 2387 (298) | 186055 (23257) | 114729 (14341) |
| Shanxi | 1313 (164) | 27 (3) | 4056 (507) | 1705 (213) | 72099 (9012) | 69989 (8749) |
| Sichuan | 1212 (151) | 109 (14) | 4522 (565) | 2321 (290) | 58289 (7286) | 88381 (11048) |
| Tianjin | 1845 (231) | 59 (7) | 7457 (932) | 2170 (271) | 120153 (15019) | 131710 (16464) |
| Tibet | 1332 (166) | 109 (14) | 4023 (503) | 2195 (274) | 138039 (17255) | 88096 (11012) |
| Xinjiang | 1087 (136) | 30 (4) | 4427 (553) | 2297 (287) | 100495 (12562) | 88096 (11012) |
| Yunnan | 1137 (142) | 178 (22) | 4235 (529) | 2249 (281) | 68623 (8578) | 89635 (11204) |
| Zhejiang | 1589 (199) | 44 (5) | 7143 (893) | 2097 (262) | 110418 (13802) | 106464 (13308) |

*The discounted cost of special education estimated for ages 6-18 years using the societal perspective.

**REFERENCES**

1. Sun B. Investigation on neonatal hearing screening status in Zhengzhou, 2009–2013. Maternal and Child Health Care of China.2015; 30(4):571-573.

2. Qiu J, Yu C, Ariyaratne TV, Foteff C, Ke Z, Sun Y, Zhang L, Qin F, Sanderson G. Cost-Effectiveness of Pediatric Cochlear Implantation in Rural China. Otology & neurotology : official publication of the American Otological Society, American Neurotology Society [and] European Academy of Otology and Neurotology.2017; 38(6):e75-e84.

3. Ning GJ, Wang XX, Liu SW, Zhu YY, Zhang BL，Zhang XS.Retrospective Investigation on Diseases Burden of Children with Community Acquired Pneumonia under 5 Years in Baiyin City of Gansu Province, 2015–2016. Chinese Journal of Vaccines and Immunization.2017; 23(1):18-21+12.

4. Liu W. Study on economic burden of bacterial meningitis in China. Beijing:Chinese Center for Disease Control and Prevention; 2016.

5. Yu W, Lu M, Wang H, Rodewald L, Ji S, Ma C, Li Y, Zheng J, Song Y, Wang M et al. Routine immunization services costs and financing in China, 2015. Vaccine.2018; 36(21):3041-3047.

6. Ministry of Education of the People's Republic of China: China Education Statistics Yearbook 2018. China Statistics Press.Beijing, 2018.

7. Ministry of Education of the People's Republic of China: China Education Expenditure Statistical Yearbook 2018. China Statistics Press.Beijing, 2018.
