# Supplementary material for: National and provincial impact and cost-effectiveness of Haemophilus influenzae type b conjugate vaccine in China: a modeling analysis
Source: BMC Med. 2021 Aug 11;19:181. doi: 10.1186/s12916-021-02049-7 (PMC8356460; doi:10.1186/s12916-021-02049-7)
Supplement: Supplementary file 4 — Additional file 4: Table S8- Summary of Hib vaccine products available in China; Table S9- Provincial vaccine coverage and weighted vaccine coverage in the private in 2017; Table S10- Regional dose-specific and weighted combined Hib vaccine coverage used for the NIP strategy; Table S11- Societal cost per dose of the National Immunization Program (NIP) in China in 2015 (2017 US$). [file 12916_2021_2049_MOESM4_ESM.docx]

**Additional file 4.** **Data sources and methods for estimating Hib vaccine coverage for each strategy and the cost of immunization delivery per dose by province**

*Hib Vaccine Availability in China*

There are currently four Hib-contained vaccine products (Monovalent Hib, DTaP-Hib, MenAC-Hib, DTaP-Hib-IPV) available in the private market for children between 2 months-5 years in China. The available vaccines, manufactured by four domestic companies and one foreign company (SANOFI PASTEUR S.A.), range in price per dose from US$ 9.1 to US$ 88.1. A 3-dose schedule or 4-dose schedule is recommended for all of the Hib vaccine products except DTaP-Hib and DTaP-Hib-IPV, which are only recommended in a 4-dose schedule. The details for each vaccine product are described in Table 1.

**Table 1. Summary of Hib vaccine products available in China**

| **Vaccine Product** | **Manufacturer** | **Eligible Population** | **Dosing Schedule** | | **Price per Dose (US$)** |
| --- | --- | --- | --- | --- | --- |
|  |  |  | **4-Dose Schedule** | **3-Dose Schedule** |  |
| Monovalent Hib | Chongqing Zhifei Biological Products Co., Ltd. | 2 mo – 5 yrs | 2, 4, 6, 18 mo | 6-12, 18 mo | 10.0 |
|  | Beijing Minhai Biological Technology Co., Ltd. | 3 mo – 5 yrs | 3,4,5, 18 mo | 6-12, 18 mo | 10.3 |
|  | SANOFI PASTEUR S.A. | 2 mo – 5 yrs | 2, 4, 6, 18 mo | 6-12, 18 mo | 14.8 |
|  | Lanzhou Institute of Biological Products Co., Ltd. | 3 mo – 5 yrs | 3,4,5, 18 mo | 6-12, 18 mo | 9.1 |
|  | Yuxi Watson Biotechnology Co., Ltd. | 2 mo – 5 yrs | 2, 4, 6, 18 mo | 6-12, 18 mo | 14.4 |
| DTaP-Hib | Beijing Minhai Biological Technology Co., Ltd. | >3 mo | 3,4,5, 18-24 mo | NA | 40.4 |
| MenAC-Hib | Chongqing Zhifei Biological Products Co., Ltd. | 2-71 mo | 2-5, 12-71 mo | 6-11, 12-71 mo | 30.9 |
| DTaP-Hib-IPV | SANOFI PASTEUR S.A. | >2 mo | 2,3.4,18 mo or 3,4,5,18 mo | NA | 88.1 |

Hib - Haemophilus influenzae type b conjugate vaccine

DTap-Hib - Diphtheria and tetanus toxoid with acellular pertussis and Hib vaccine

MenAC-Hib - Meningococcal Groups Aand C and Haemophilus b conjugate vaccine

DTaP-Hib-IPV - Diphtheria and tetanus toxoid with acellular pertussis, Hib and IPV vaccine

*Hib Vaccine Coverage*

For *status quo* strategy, provincial coverage rates for each dose of Hib vaccine in the private market were estimated using data from the China CDC and a 2019 facility-based survey of more than 6,000 children in 10 provinces in China.[1] To estimate the Hib vaccine coverage by dose in each province, we used the following formula:

$$C_{p,j}=\frac{D_{p}w_{j}}{N_{p}j}$$

where *C_p,j_* is the coverage for *j* total doses (1, 2, 3, or 4 doses) in province *p*. *D_p_* is the total number of doses delivered in province *p* obtained from China CDC, *w_j_* is the proportion of children receiving *j* total doses obtained from the survey conducted in 10 provinces, and *N_p_* is the number of neonates in the province. We estimated the doses-specific coverage using survey data from only children older than 18 months of age because the minimum age for a child to be fully vaccinated in the 4-dose schedule is 18 months old in China. For the 21 provinces not included in the facility-based survey, data from neighboring provinces with similar levels of economic development were used.

For the herd immunity sensitivity scenario analysis, we estimated the combined vaccine effect for direct and indirect effects by estimating the weighted vaccine coverage using the following formula:

$$C_{w}= \sum_{j} C_{j}\frac{E_{j}}{E_{3}}$$

where *C_w_* is the weighted vaccine coverage, *C_j_* is the coverage for *j* total doses, and *E_j_* is the vaccine efficacy for *j* total doses. The dose-specific vaccine efficacies used in the model from Griffiths et al. 2012[2] were: 59% for 1 dose, 92% for 2 doses, and 93% for 3 and 4 doses. See Table 2 for provincial dose-specific coverage in the private market. The impact of including herd immunity in the model was assessed at different vaccine coverage levels using the following regression model developed by Wahl et al.[3]:

$${VEC}_{combined}= \left\{ \begin{aligned} C_{w}\times E , &C_{w}<10\% or C_{w}\geq98\% \\ \left( C_{w}\times2.428 \right)-0.138, &10\% \leq C_{w} < 40\% \\ (C_{w}*2.428) - ((C_{w}-0.40)*2.257) - 0.138, &40\% \leq C_{w} < 98\% \end{aligned} \right.$$

where *VEC_combined_* is the vaccine effective coverage of the combined direct and herd immunity effect, *C_w_* is the weighted vaccine coverage of all doses, and *E* is the combined efficacy against death and cases (95% for Hib vaccine).[4] When *C_w_* is <10% and ≥98%, *VEC_combined_* equals the vaccine effective coverage of direct effects only.

**Table 2. Provincial vaccine coverage and weighted vaccine coverage in the private in 2017**

| **Province** | **Hib Vaccine 1 Dose Coverage** | **Hib Vaccine 2 Dose Coverage** | **Hib Vaccine**  **3 Dose Coverage** | **Hib Vaccine**  **4 Dose Coverage** | **Weighted Vaccine Coverage** |
| --- | --- | --- | --- | --- | --- |
| Anhui | 0.083 | 0.057 | 0.132 | 0.120 | 0.361 |
| Beijing | 0.152 | 0.089 | 0.025 | 0.177 | 0.386 |
| Chongqing | 0.128 | 0.055 | 0.132 | 0.206 | 0.473 |
| Fujian | 0.114 | 0.066 | 0.071 | 0.086 | 0.295 |
| Gansu | 0.020 | 0.012 | 0.012 | 0.025 | 0.062 |
| Guangdong | 0.195 | 0.113 | 0.121 | 0.147 | 0.503 |
| Guangxi | 0.090 | 0.052 | 0.099 | 0.113 | 0.320 |
| Guizhou | 0.064 | 0.037 | 0.071 | 0.080 | 0.229 |
| Hainan | 0.114 | 0.066 | 0.071 | 0.086 | 0.295 |
| Hebei | 0.068 | 0.049 | 0.064 | 0.078 | 0.233 |
| Heilongjiang | 0.353 | 0.059 | 0.029 | 0.010 | 0.321 |
| Henan | 0.051 | 0.049 | 0.154 | 0.186 | 0.421 |
| Hubei | 0.139 | 0.059 | 0.143 | 0.222 | 0.512 |
| Hunan | 0.076 | 0.032 | 0.077 | 0.121 | 0.278 |
| Inner Mongolia | 0.037 | 0.023 | 0.023 | 0.046 | 0.114 |
| Jiangsu | 0.019 | 0.005 | 0.021 | 0.094 | 0.132 |
| Jiangxi | 0.088 | 0.060 | 0.139 | 0.126 | 0.381 |
| Jilin | 0.257 | 0.043 | 0.021 | 0.007 | 0.234 |
| Liaoning | 0.260 | 0.043 | 0.022 | 0.007 | 0.236 |
| Ningxia | 0.024 | 0.015 | 0.015 | 0.030 | 0.075 |
| Qinghai | 0.016 | 0.010 | 0.010 | 0.020 | 0.050 |
| Shaanxi | 0.019 | 0.018 | 0.058 | 0.070 | 0.158 |
| Shandong | 0.098 | 0.070 | 0.093 | 0.113 | 0.338 |
| Shanghai | 0.107 | 0.031 | 0.121 | 0.539 | 0.758 |
| Shanxi | 0.019 | 0.018 | 0.058 | 0.070 | 0.158 |
| Sichuan | 0.138 | 0.059 | 0.141 | 0.220 | 0.506 |
| Tianjin | 0.221 | 0.129 | 0.037 | 0.258 | 0.563 |
| Tibet | 0.008 | 0.005 | 0.005 | 0.009 | 0.024 |
| Xinjiang | 0.007 | 0.004 | 0.004 | 0.008 | 0.021 |
| Yunnan | 0.070 | 0.040 | 0.077 | 0.087 | 0.248 |
| Zhejiang | 0.064 | 0.019 | 0.072 | 0.321 | 0.452 |

For the NIP, the regional 4-dose diphtheria-tetanus-pertussis vaccine (DTP) coverage was used as a proxy for Hib vaccine coverage because of their similar dosing schedules in China (See Table 3). Regional 4-dose DTP coverage was obtained from a China CDC survey conducted in 2012.[5]

**Table 3. Regional dose-specific and weighted combined Hib vaccine coverage used for the NIP strategy**

| **Dose** | **East Region**  **Base Case (Range)** | **Central Region**  **Base Case (Range)** | **West Region**  **Base Case (Range)** |
| --- | --- | --- | --- |
| 1 Dose | 0.0009 (0.0000 - 0.0009) | 0.0009 (0.0000 - 0.0009) | 0.0009 (0.0000 - 0.0009) |
| 2 Doses | 0.0013 (0.0000 - 0.0013) | 0.0013 (0.0000 - 0.0013) | 0.0066 (0.0000 - 0.0066) |
| 3 Doses | 0.0346 (0.0000 - 0.0346) | 0.0459 (0.0000 - 0.0459) | 0.1458 (0.0000 - 0.1458) |
| 4 Doses | 0.9598 (0.8638 - 0.9598) | 0.9485 (0.8537 - 0.9485) | 0.8433 (0.7590 - 0.8433) |
| Weighted Vaccine Coverage | 0.9963 (0.8967–0.9963) | 0.9963 (0.8967-0.9963) | 0.9962 (0.8966-0.9962) |

Vaccine coverage was assumed to have a triangular distribution for deterministic and probabilistic sensitivity analysis.

*Vaccine Program Costs*

The societal cost of the Hib vaccine program was estimated using regional vaccine program data from a 2015 survey conducted by China CDC in 15 provinces (See Table 4).[6] The governmental cost of routine immunization included the cost of vaccines, wastage, personnel, cold chain, surveillance, communication activities, training, and supervision at the national and provincial levels. We assumed the wastage rate of Hib vaccine, regardless of the product used, was 5% (range 0-10%) following recommendations from WHO for similar vaccines. The household cost of vaccine-seeking included the cost of transportation and caregiver productivity loss. For the 16 provinces not included in the surveys by China CDC, regional government and household cost estimates were used. The per dose cost of serious adverse reactions was estimated using the cost of abnormal medical examination (US$ 903 or￥6,142) from the 2018 China Health and Family Planning Statistical Yearbook,[7] and the incidence of Hib vaccine adverse events following immunization was obtained from the Adverse Events Following Immunization System (AEFIs) of National Immunization Program Information Management System.[8]

**Table 4. Societal cost per dose of the National Immunization Program (NIP) in China in 2015 (2017 US$)**

| **Province or Region** | **Government Vaccine Program Cost per Dose** | | | | | | | | **Household Cost per Dose** | **Total Cost per Dose** |
| --- | --- | --- | --- | --- | --- | --- | --- | --- | --- | --- |
|  | **Personnel** | **Office**  **Building** | **Cold**  **Chain** | **Surveillance** | **Communication** | **Training** | **Supervision** | **Other** |  |  |
| Beijing | 2.6 | 0.2 | 0.2 | 0.1 | 0.1 | 0.0 | 0.0 | 0.1 | 10.3 | 13.6 |
| Hebei | 1.5 | 0.1 | 0.3 | 0.1 | 0.3 | 0.1 | 0.0 | 0.1 | 3.8 | 6.3 |
| Shanxi | 1.9 | 0.1 | 0.2 | 0.1 | 0.1 | 0.1 | 0.0 | 0.0 | 3.5 | 6.0 |
| Heilongjiang | 2.3 | 0.1 | 0.5 | 0.2 | 0.5 | 0.2 | 0.2 | 0.2 | 6.3 | 10.4 |
| Jiangsu | 3.7 | 0.2 | 0.2 | 0.3 | 0.4 | 0.1 | 0.1 | 0.2 | 6.2 | 11.2 |
| Zhejiang | 2.8 | 0.2 | 0.1 | 0.2 | 0.2 | 0.1 | 0.0 | 0.1 | 6.2 | 9.9 |
| Anhui | 1.6 | 0.1 | 0.1 | 0.1 | 0.1 | 0.1 | 0.1 | 0.1 | 4.7 | 7.0 |
| Jiangxi | 1.3 | 0.1 | 0.1 | 0.1 | 0.1 | 0.1 | 0.1 | 0.0 | 4.8 | 6.6 |
| Shandong | 1.9 | 0.1 | 0.2 | 0.2 | 0.2 | 0.1 | 0.0 | 0.1 | 4.6 | 7.4 |
| Hunan | 2.2 | 0.1 | 0.4 | 0.2 | 0.3 | 0.2 | 0.1 | 0.1 | 4.7 | 8.4 |
| Guangdong | 1.6 | 0.2 | 0.2 | 0.2 | 0.1 | 0.1 | 0.0 | 0.1 | 6.1 | 8.7 |
| Guangxi | 2.5 | 0.1 | 0.2 | 0.2 | 0.3 | 0.1 | 0.1 | 0.1 | 7.8 | 11.2 |
| Chongqing | 2.9 | 0.1 | 0.2 | 0.1 | 0.2 | 0.1 | 0.1 | 0.1 | 5.9 | 9.6 |
| Sichuan | 1.9 | 0.1 | 0.5 | 0.1 | 0.4 | 0.3 | 0.1 | 0.1 | 5.8 | 9.4 |
| Gansu | 2.9 | 0.2 | 0.4 | 0.2 | 0.4 | 0.1 | 0.1 | 0.4 | 3.5 | 8.2 |
| **East*** | 2.5 | 0.2 | 0.2 | 0.2 | 0.2 | 0.1 | 0.0 | 0.1 | 6.7 | 10.2 |
| **Central*** | 1.8 | 0.1 | 0.3 | 0.1 | 0.2 | 0.1 | 0.1 | 0.1 | 4.6 | 7.5 |
| **West*** | 2.6 | 0.1 | 0.3 | 0.1 | 0.3 | 0.2 | 0.1 | 0.2 | 5.7 | 9.6 |
| **Average national costs** | 2.2 | 0.1 | 0.2 | 0.2 | 0.2 | 0.1 | 0.1 | 0.1 | 5.6 | 8.9 |

* For the 16 provinces not included in the China CDC survey, regional routine immunization cost estimates were used.

**REFERENCES**

1. Lai X, Rong H, Ma X, Hou Z, Li S, Jing R, Zhang H, Peng Z, Feng L, Fang H. Willingness to Pay for Seasonal Influenza Vaccination among Children, Chronic Disease Patients, and the Elderly in China: A National Cross-Sectional Survey. Vaccines (Basel).2020; 8(3).

2. Griffiths UK, Clark A, Gessner B, Miners A, Sanderson C, Sedyaningsih ER, Mulholland KE. Dose-specific efficacy of Haemophilus influenzae type b conjugate vaccines: a systematic review and meta-analysis of controlled clinical trials. Epidemiol Infect.2012; 140(8):1343-1355.

3. Wahl B, O'Brien KL, Greenbaum A, Majumder A, Liu L, Chu Y, Lukšić I, Nair H, McAllister DA, Campbell H et al. Burden of Streptococcus pneumoniae and Haemophilus influenzae type b disease in children in the era of conjugate vaccines: global, regional, and national estimates for 2000-15. Lancet Glob Health.2018; 6(7):e744-e757.

4. Watt JP, Wolfson LJ, O'Brien KL, Henkle E, Deloria-Knoll M, McCall N, et al. Burden of disease caused by Haemophilus influenzae type b in children younger than 5 years: global estimates. The Lancet. 2009;374(9693):903-11.

5. Cao L, Wang HQ, Zheng JS, Yuan P，Cao LS, Zhang GM. National Immunization Coverage Survey in China after Integrated more Vaccines into EPI Since 2008. Chinese journal of vaccines and immunization.2012; 18(05):419-424+478.

6. Yu W, Lu M, Wang H, Rodewald L, Ji S, Ma C, Li Y, Zheng J, Song Y, Wang M et al. Routine immunization services costs and financing in China, 2015. Vaccine.2018; 36(21):3041-3047.

7. National Health and Family Planning Commission of China: China Health

and Family Planning Statistical Yearbook 2018. Chinese Academy of Medical

Sciences & Peking Union Medical College Press. Beijing, 2018.

8. Li K, Zhang L, Ye J, Ji S，Yu W, Cao L. Surveillance of adverse events following immunization in China, 2017. Chinese Journal of Vaccines and Immunization.2020; 26(1):9-18.
