# Supplementary material for: National and provincial impact and cost-effectiveness of Haemophilus influenzae type b conjugate vaccine in China: a modeling analysis
Source: BMC Med. 2021 Aug 11;19:181. doi: 10.1186/s12916-021-02049-7 (PMC8356460; doi:10.1186/s12916-021-02049-7)
Supplement: Supplementary file 5 — Additional file 5: Table S12- Provincial disease burden and economic supplemental results; Table S13- Provincial discounted economic costs of Hib disease and vaccine program costs (2017 US$) for each vaccination strategy from the societal perspective. [file 12916_2021_2049_MOESM5_ESM.docx]

**Additional file 5. Provincial disease burden and economic supplemental results**

**Table 1. Provincial syndrome**-**specific cases and deaths averted for each vaccination strategy**

| **Province and Region** | **Hib Cases Averted** | | | | | | **Hib Deaths Averted** | | | |
| --- | --- | --- | --- | --- | --- | --- | --- | --- | --- | --- |
|  | **Inpatient Pneumonia** | **Outpatient Pneumonia** | **Meningitis** | **NPNM** | **Meningitis Sequelae** | **Total Cases Averted** | **Inpatient Pneumonia** | **Meningitis** | **NPNM** | **Total Deaths Averted** |
| Anhui | 1637 | 7680 | 178 | 60 | 21 | 9555 | 74 | 8 | 0 | 82 |
| Beijing | 577 | 2710 | 26 | 9 | 3 | 3322 | 13 | 1 | 0 | 14 |
| Chongqing | 667 | 3129 | 49 | 17 | 6 | 3862 | 24 | 2 | 0 | 27 |
| Fujian | 1167 | 5475 | 92 | 31 | 11 | 6765 | 44 | 4 | 0 | 48 |
| Gansu | 1148 | 5389 | 310 | 105 | 36 | 6952 | 116 | 14 | 0 | 130 |
| Guangdong | 3621 | 16,993 | 234 | 79 | 27 | 20,928 | 113 | 10 | 0 | 123 |
| Guangxi | 1743 | 8181 | 148 | 50 | 17 | 10,123 | 85 | 7 | 0 | 91 |
| Guizhou | 1357 | 6370 | 101 | 34 | 12 | 7862 | 78 | 4 | 0 | 83 |
| Hainan | 338 | 1588 | 58 | 20 | 7 | 2004 | 41 | 3 | 0 | 44 |
| Hebei | 2847 | 13,358 | 592 | 199 | 69 | 16,996 | 160 | 26 | 0 | 186 |
| Heilongjiang | 681 | 3197 | 90 | 30 | 12 | 4000 | 25 | 4 | 0 | 29 |
| Henan | 2241 | 10,517 | 324 | 109 | 38 | 13,191 | 99 | 14 | 0 | 113 |
| Hubei | 980 | 4600 | 58 | 20 | 7 | 5658 | 34 | 3 | 0 | 37 |
| Hunan | 1819 | 8535 | 80 | 27 | 9 | 10,461 | 49 | 4 | 0 | 52 |
| Inner Mongolia | 848 | 3981 | 161 | 54 | 19 | 5045 | 53 | 7 | 0 | 60 |
| Jiangsu | 2248 | 10,547 | 82 | 28 | 10 | 12,904 | 29 | 4 | 0 | 33 |
| Jiangxi | 1250 | 5867 | 266 | 90 | 31 | 7473 | 134 | 12 | 0 | 146 |
| Jilin | 709 | 3328 | 111 | 38 | 13 | 4186 | 30 | 5 | 0 | 35 |
| Liaoning | 1045 | 4902 | 44 | 15 | 5 | 6006 | 14 | 2 | 0 | 16 |
| Ningxia | 314 | 1473 | 59 | 20 | 7 | 1865 | 32 | 3 | 0 | 35 |
| Qinghai | 307 | 1442 | 105 | 35 | 12 | 1889 | 55 | 5 | 0 | 60 |
| Shaanxi | 1208 | 5669 | 286 | 96 | 33 | 7260 | 114 | 13 | 0 | 126 |
| Shandong | 2392 | 11,227 | 160 | 54 | 19 | 13,833 | 38 | 7 | 0 | 46 |
| Shanghai | 220 | 1034 | 10 | 3 | 1 | 1268 | 5 | 0 | 0 | 6 |
| Shanxi | 1191 | 5589 | 262 | 88 | 30 | 7130 | 96 | 12 | 0 | 107 |
| Sichuan | 1930 | 9057 | 194 | 65 | 23 | 11,246 | 139 | 9 | 0 | 148 |
| Tianjin | 234 | 1098 | 18 | 6 | 2 | 1356 | 9 | 1 | 0 | 10 |
| Tibet | 291 | 1365 | 30 | 10 | 3 | 1695 | 74 | 6 | 0 | 80 |
| Xinjiang | 1474 | 6916 | 268 | 90 | 31 | 8748 | 359 | 32 | 0 | 391 |
| Yunnan | 2350 | 11,029 | 353 | 119 | 41 | 13,851 | 297 | 16 | 0 | 312 |
| Zhejiang | 1427 | 6696 | 78 | 26 | 9 | 8228 | 29 | 3 | 0 | 32 |
| East | 16,116 | 75,627 | 1396 | 471 | 162 | 93,609 | 496 | 62 | 0 | 558 |
| Central | 10,508 | 49,134 | 1369 | 462 | 161 | 61,654 | 540 | 61 | 0 | 601 |
| West | 13,638 | 64,000 | 2065 | 696 | 239 | 80,398 | 1426 | 117 | 1 | 1544 |
| National | 40,261 | 188,941 | 4829 | 1628 | 562 | 235,659 | 2462 | 240 | 2 | 2704 |

Rows and columns may not sum to the total due to rounding.

**Table 2. Provincial discounted economic costs of Hib disease and vaccine program costs (2017 US$) for each vaccination strategy from the societal perspective**

| **Province and Region** | **Economic Costs of Hib Disease***  **(US$ in thousands)** | | | **Vaccine Program Costs**  **(US$ in thousands)** | | | **Total Costs**  **(US$ in thousands)** | | |
| --- | --- | --- | --- | --- | --- | --- | --- | --- | --- |
|  | **Status Quo** | **NIP** | **Difference** | **Status Quo** | **NIP** | **Difference** | **Status Quo** | **NIP** | **Difference** |
| Anhui | 12,822.3 | 580.4 | 12,242.0 | 17,018.0 | 62,449.3 | 45,431.3 | 29,840.3 | 63,029.7 | 33,189.4 |
| Beijing | 4,774.3 | 235.4 | 4,538.8 | 6,551.6 | 23,248.3 | 16,696.7 | 11,325.9 | 23,483.7 | 12,157.8 |
| Chongqing | 5,046.9 | 311.1 | 4,735.8 | 10,124.8 | 26,620.2 | 16,495.4 | 15,171.7 | 26,931.3 | 11,759.6 |
| Fujian | 9,121.6 | 289.3 | 8,832.2 | 10,700.7 | 52,500.9 | 41,800.1 | 19,822.3 | 52,790.2 | 32,967.9 |
| Gansu | 17,401.4 | 793.8 | 16,607.6 | 1,249.3 | 26,634.7 | 25,385.3 | 18,650.7 | 27,428.5 | 8,777.8 |
| Guangdong | 28,689.6 | 1,896.0 | 26,793.5 | 49,920.0 | 143,605.4 | 93,685.4 | 78,609.6 | 145,501.4 | 66,891.8 |
| Guangxi | 12,272.6 | 468.0 | 11,804.5 | 17,276.5 | 70,169.0 | 52,892.5 | 29,549.1 | 70,637.0 | 41,087.9 |
| Guizhou | 12,435.1 | 481.5 | 11,953.6 | 9,094.7 | 51,757.8 | 42,663.1 | 21,529.8 | 52,239.3 | 30,709.5 |
| Hainan | 6,226.6 | 370.8 | 5,855.8 | 2,446.9 | 11,991.3 | 9,544.5 | 8,673.5 | 12,362.1 | 3,688.6 |
| Hebei | 29,070.7 | 1,471.1 | 27,599.6 | 11,781.9 | 69,550.7 | 57,768.8 | 40,852.6 | 71,021.8 | 30,169.2 |
| Heilongjiang | 4,637.1 | 220.8 | 4,416.3 | 2,758.0 | 18,145.8 | 15,387.7 | 7,395.1 | 18,366.6 | 10,971.5 |
| Henan | 17,258.2 | 702.5 | 16,555.7 | 38,884.0 | 112,983.8 | 74,099.8 | 56,142.2 | 113,686.3 | 57,544.1 |
| Hubei | 5,652.9 | 213.9 | 5,439.0 | 20,452.8 | 51,121.4 | 30,668.6 | 26,105.7 | 51,335.3 | 25,229.6 |
| Hunan | 7,985.5 | 34.3 | 7,951.2 | 16,055.8 | 73,901.1 | 57,845.3 | 24,041.3 | 73,935.4 | 49,894.1 |
| Inner Mongolia | 9,386.4 | 442.3 | 8,944.1 | 1,527.0 | 17,549.5 | 16,022.5 | 10,913.4 | 17,991.8 | 7,078.4 |
| Jiangsu | 9,842.9 | 50.5 | 9,792.4 | 8,901.5 | 75,363.4 | 66,461.9 | 18,744.4 | 75,413.9 | 56,669.5 |
| Jiangxi | 19,050.6 | 1,263.3 | 17,787.3 | 12,639.4 | 43,959.1 | 31,319.7 | 31,690.0 | 45,222.4 | 13,532.4 |
| Jilin | 5,564.5 | 288.7 | 5,275.8 | 1,417.0 | 12,791.7 | 11,374.8 | 6,981.5 | 13,080.4 | 6,098.9 |
| Liaoning | 4,060.5 | 84.3 | 3,976.2 | 2,936.3 | 26,346.2 | 23,409.9 | 6,996.8 | 26,430.5 | 19,433.7 |
| Ningxia | 4,342.2 | 194.2 | 4,147.9 | 455.4 | 7,970.8 | 7,515.4 | 4,797.6 | 8,165.0 | 3,367.4 |
| Qinghai | 7,223.5 | 358.3 | 6,865.2 | 254.0 | 6,670.5 | 6,416.5 | 7,477.5 | 7,028.8 | -448.7 |
| Shaanxi | 17,441.2 | 841.1 | 16,600.1 | 4,925.9 | 37,117.5 | 32,191.6 | 22,367.1 | 37,958.6 | 15,591.5 |
| Shandong | 12,535.7 | 110.8 | 12,424.9 | 29,125.4 | 118,389.2 | 89,263.8 | 41,661.1 | 118,500.0 | 76,838.9 |
| Shanghai | 1,659.9 | 195.4 | 1,464.5 | 12,603.5 | 18,513.3 | 5,909.8 | 14,263.4 | 18,708.7 | 4,445.3 |
| Shanxi | 13,329.6 | 2,441.6 | 10,888.1 | 3,529.2 | 27,303.6 | 23,774.4 | 16,858.8 | 29,745.2 | 12,886.4 |
| Sichuan | 20,360.5 | 1,616.3 | 18,744.1 | 29,196.1 | 71,663.9 | 42,467.8 | 49,556.6 | 73,280.2 | 23,723.6 |
| Tianjin | 2,415.1 | 203.2 | 2,211.8 | 4,279.3 | 10,401.7 | 6,122.4 | 6,694.4 | 10,604.9 | 3,910.5 |
| Tibet | 8,421.3 | 421.5 | 7,999.8 | 87.1 | 4,831.9 | 4,744.8 | 8,508.4 | 5,253.4 | -3,255.0 |
| Xinjiang | 41,955.9 | 2,033.5 | 39,922.4 | 527.8 | 33,622.0 | 33,094.2 | 42,483.7 | 35,655.5 | -6,828.2 |
| Yunnan | 39,148.6 | 2,364.0 | 36,784.6 | 10,307.3 | 53,981.0 | 43,673.7 | 49,455.9 | 56,345.0 | 6,889.1 |
| Zhejiang | 8,071.1 | 322.6 | 7,748.6 | 24,528.3 | 60,482.4 | 35,954.2 | 32,599.4 | 60,805.0 | 28,205.6 |
| **East** | 116,467.9 | 5,229.6 | 111,238.3 | 163,775.4 | 610,392.7 | 446,617.3 | 280,243.3 | 615,622.3 | 335,379.0 |
| **West** | 86,300.8 | 5,745.5 | 80,555.3 | 112,754.2 | 402,655.8 | 289,901.6 | 199,055.0 | 408,401.3 | 209,346.3 |
| **Central** | 195,435.5 | 10,325.6 | 185,109.9 | 85,025.9 | 408,588.7 | 323,562.7 | 280,461.4 | 418,914.3 | 138,452.9 |
| **National** | 398,204.2 | 21,300.6 | 376,903.5 | 361,555.5 | 1,421,637.1 | 1,060,081.6 | 759,759.7 | 1,442,937.7 | 683,178.0 |

*Includes the cost of treatment for Hib disease, Hib meningitis sequelae, and lost productivity due to disability and premature death
