# Supplementary material for: National and provincial impact and cost-effectiveness of Haemophilus influenzae type b conjugate vaccine in China: a modeling analysis
Source: BMC Med. 2021 Aug 11;19:181. doi: 10.1186/s12916-021-02049-7 (PMC8356460; doi:10.1186/s12916-021-02049-7)
Supplement: Supplementary file 6 — Additional file 6: Table S14- Incremental cost-effectiveness ratios of including Hib vaccine in the NIP in 2017 (RMB); Table S15- Incremental cost-effectiveness ratios of including Hib vaccine in the NIP in 2017 (US$); Table S16- Hib cases and deaths averted by province and region when including herd immunity effects. [file 12916_2021_2049_MOESM6_ESM.docx]

**Additional file 6.** **Incremental cost-effectiveness ratios of including Hib vaccine in the NIP for the base case and herd immunity sensitivity analysis**

**Table 1. Incremental cost-effectiveness ratios of including Hib vaccine in the NIP in 2017 (RMB)**

| **Province and Region** | **Base Case^*^** | | | | **Herd Immunity Scenario^*^** | | | |
| --- | --- | --- | --- | --- | --- | --- | --- | --- |
|  | **Cost per Case Averted** | **Cost per Death Averted** | **Cost per QALY gained** | **Rank** | **Cost per Case Averted** | **Cost per Death Averted** | **Cost per QALY gained** | **Rank** |
| Anhui | 23,620 | 2,760,427 | 85,870 | **18** | 84,536 | 9,879,695 | 307,332 | **19** |
| Beijing | 24,887 | 5,725,769 | 177,827 | **26** | 124,415 | 28,624,753 | 889,010 | **30** |
| Chongqing | 20,708 | 3,012,199 | 94,291 | **19** | 134,166 | 19,515,709 | 610,904 | **24** |
| Fujian | 33,138 | 4,683,378 | 146,437 | **23** | 66,900 | 9,454,891 | 295,630 | **18** |
| Gansu | 8,585 | 459,011 | 14,339 | **5** | 8,587 | 459,087 | 14,341 | **4** |
| Guangdong | 21,735 | 3,685,778 | 114,793 | **22** | 139,467 | 23,650,516 | 736,595 | **26** |
| Guangxi | 27,601 | 3,062,769 | 97,031 | **20** | 66,156 | 7,340,949 | 232,568 | **16** |
| Guizhou | 26,560 | 2,521,652 | 81,380 | **16** | 40,386 | 3,834,391 | 123,746 | **13** |
| Hainan | 12,515 | 573,476 | 18,528 | **6** | 38,629 | 1,770,067 | 57,186 | **10** |
| Hebei | 12,071 | 1,101,479 | 33,263 | **12** | 21,179 | 1,932,687 | 58,363 | **11** |
| Heilongjiang | 18,651 | 2,572,607 | 78,303 | **15** | 47,453 | 6,634,926 | 199,231 | **15** |
| Henan | 29,664 | 3,460,921 | 105,186 | **21** | 180,485 | 21,057,361 | 639,988 | **25** |
| Hubei | 30,322 | 4,636,789 | 147,034 | **24** | 172,444 | 26,479,565 | 836,249 | **27** |
| Hunan | 32,434 | 6,507,443 | 204,445 | **28** | 57,247 | 11,485,974 | 360,857 | **20** |
| Inner Mongolia | 9,541 | 800,843 | 24,619 | **9** | 10,363 | 869,825 | 26,739 | **7** |
| Jiangsu | 29,862 | 11,623,336 | 345,802 | **31** | 32,428 | 12,622,206 | 375,519 | **22** |
| Jiangxi | 12,313 | 629,381 | 19,978 | **7** | 87,404 | 4,467,596 | 141,811 | **14** |
| Jilin | 9,908 | 1,183,758 | 35,569 | **14** | 17,328 | 2,070,265 | 62,206 | **12** |
| Liaoning | 22,004 | 8,385,295 | 247,203 | **29** | 32,914 | 12,542,922 | 369,772 | **21** |
| Ningxia | 12,278 | 658,708 | 21,016 | **8** | 12,278 | 658,717 | 21,017 | **6** |
| Qinghai | Cost-saving | Cost-saving | Cost-saving | **3** | Cost-saving | Cost-saving | Cost-saving | **3** |
| Shaanxi | 14,604 | 838,619 | 26,227 | **11** | 18,656 | 1,071,257 | 33,503 | **9** |
| Shandong | 37,772 | 11,476,929 | 334,769 | **30** | 97,554 | 29,641,523 | 864,610 | **28** |
| Shanghai | 23,843 | 5,282,720 | 164,129 | **25** | 129,109 | 28,605,446 | 888,745 | **29** |
| Shanxi | 12,290 | 816,759 | 25,339 | **10** | 15,562 | 1,034,200 | 32,085 | **8** |
| Sichuan | 14,345 | 1,089,156 | 35,069 | **13** | 113,676 | 8,631,056 | 277,902 | **17** |
| Tianjin | 19,618 | 2,659,036 | 83,493 | **17** | 136,083 | 18,445,115 | 579,168 | **23** |
| Tibet | Cost-saving | Cost-saving | Cost-saving | **1** | Cost-saving | Cost-saving | Cost-saving | **1** |
| Xinjiang | Cost-saving | Cost-saving | Cost-saving | **2** | Cost-saving | Cost-saving | Cost-saving | **2** |
| Yunnan | 3,382 | 149,974 | 4,887 | **4** | 13,513 | 599,232 | 19,525 | **5** |
| Zhejiang | 23,311 | 5,929,620 | 179,924 | **27** | 139,909 | 35,588,306 | 1,079,863 | **31** |
| **East** | 24,363 | 4,085,498 | 124,908 |  | 57,202 | 9,360,276 | 284,750 |  |
| **West** | 23,089 | 2,366,728 | 73,540 |  | 65,215 | 6,599,175 | 204,557 |  |
| **Central** | 11,710 | 609,780 | 19,708 |  | 22,082 | 1,005,723 | 32,556 |  |
| **National** | 19,713 | 1,718,266 | 54,406 |  | 43,707 | 3,183,696 | 101,344 |  |

^*^The base case analysis assumed no herd immunity effects in any province, and the herd immunity scenario included indirect effects based on the province vaccine coverage.

**Table 2. Incremental cost-effectiveness ratios of including Hib vaccine in the NIP in 2017 (US$)**

| **Province and Region** | **Base Case^*^** | | | | **Herd Immunity Scenario^*^** | | | |
| --- | --- | --- | --- | --- | --- | --- | --- | --- |
|  | **Cost per Case Averted** | **Cost per Death Averted** | **Cost per QALY gained** | **Rank** | **Cost per Case Averted** | **Cost per Death Averted** | **Cost per QALY gained** | **Rank** |
| Anhui | 3,473 | 405,945 | 12,628 | **18** | 12,432 | 1,452,896 | 45,196 | **19** |
| Beijing | 3,660 | 842,025 | 26,151 | **26** | 18,296 | 4,209,523 | 130,737 | **30** |
| Chongqing | 3,045 | 442,970 | 13,866 | **19** | 19,730 | 2,869,957 | 89,839 | **24** |
| Fujian | 4,873 | 688,732 | 21,535 | **23** | 9,838 | 1,390,425 | 43,475 | **18** |
| Gansu | 1,263 | 67,502 | 2,109 | **5** | 1,263 | 67,513 | 2,109 | **4** |
| Guangdong | 3,196 | 542,026 | 16,881 | **22** | 20,510 | 3,478,017 | 108,323 | **26** |
| Guangxi | 4,059 | 450,407 | 14,269 | **20** | 9,729 | 1,079,551 | 34,201 | **16** |
| Guizhou | 3,906 | 370,831 | 11,968 | **16** | 5,939 | 563,881 | 18,198 | **13** |
| Hainan | 1,840 | 84,335 | 2,725 | **6** | 5,681 | 260,304 | 8,410 | **10** |
| Hebei | 1,775 | 161,982 | 4,892 | **12** | 3,115 | 284,219 | 8,583 | **11** |
| Heilongjiang | 2,743 | 378,325 | 11,515 | **15** | 6,978 | 975,724 | 29,299 | **15** |
| Henan | 4,362 | 508,959 | 15,469 | **21** | 26,542 | 3,096,671 | 94,116 | **25** |
| Hubei | 4,459 | 681,881 | 21,623 | **24** | 25,359 | 3,894,054 | 122,978 | **27** |
| Hunan | 4,770 | 956,977 | 30,066 | **28** | 8,419 | 1,689,114 | 53,067 | **20** |
| Inner Mongolia | 1,403 | 117,771 | 3,620 | **9** | 1,524 | 127,915 | 3,932 | **7** |
| Jiangsu | 4,391 | 1,709,314 | 50,853 | **31** | 4,769 | 1,856,207 | 55,223 | **22** |
| Jiangxi | 1,811 | 92,556 | 2,938 | **7** | 12,854 | 656,999 | 20,855 | **14** |
| Jilin | 1,457 | 174,082 | 5,231 | **14** | 2,548 | 304,451 | 9,148 | **12** |
| Liaoning | 3,236 | 1,233,132 | 36,353 | **29** | 4,840 | 1,844,547 | 54,378 | **21** |
| Ningxia | 1,806 | 96,869 | 3,091 | **8** | 1,806 | 96,870 | 3,091 | **6** |
| Qinghai | Cost-saving | Cost-saving | Cost-saving | **3** | Cost-saving | Cost-saving | Cost-saving | **3** |
| Shaanxi | 2,148 | 123,326 | 3,857 | **11** | 2,744 | 157,538 | 4,927 | **9** |
| Shandong | 5,555 | 1,687,784 | 49,231 | **30** | 14,346 | 4,359,048 | 127,149 | **28** |
| Shanghai | 3,506 | 776,871 | 24,137 | **25** | 18,987 | 4,206,683 | 130,698 | **29** |
| Shanxi | 1,807 | 120,112 | 3,726 | **10** | 2,288 | 152,088 | 4,718 | **8** |
| Sichuan | 2,110 | 160,170 | 5,157 | **13** | 16,717 | 1,269,273 | 40,868 | **17** |
| Tianjin | 2,885 | 391,035 | 12,278 | **17** | 20,012 | 2,712,517 | 85,172 | **23** |
| Tibet | Cost-saving | Cost-saving | Cost-saving | **1** | Cost-saving | Cost-saving | Cost-saving | **1** |
| Xinjiang | Cost-saving | Cost-saving | Cost-saving | **2** | Cost-saving | Cost-saving | Cost-saving | **2** |
| Yunnan | 497 | 22,055 | 719 | **4** | 1,987 | 88,122 | 2,871 | **5** |
| Zhejiang | 3,428 | 872,003 | 26,459 | **27** | 20,575 | 5,233,574 | 158,803 | **31** |
| **East** | 3,583 | 600,809 | 18,369 |  | 8,412 | 1,376,511 | 41,875 |  |
| **West** | 3,396 | 348,048 | 10,815 |  | 9,590 | 970,467 | 30,082 |  |
| **Central** | 1,722 | 89,674 | 2,898 |  | 3,247 | 147,900 | 4,788 |  |
| **National** | 2,899 | 252,686 | 8,001 |  | 6,427 | 468,191 | 14,903 |  |

^*^The base case analysis assumed no herd immunity effects in any province, and the herd immunity scenario included indirect effects based on the province vaccine coverage.

**Table 3. Hib cases and deaths averted by province and region when including herd immunity effects**

| **Province and Region** | **Hib Cases Averted** | | | | | **Hib Deaths Averted** | | | **Total Hib Cases and Deaths Averted** | |
| --- | --- | --- | --- | --- | --- | --- | --- | --- | --- | --- |
|  | **Inpatient Pneumonia** | **Outpatient Pneumonia** | **Meningitis** | **Inpatient NPNM** | **Meningitis Sequelae** | **Inpatient Pneumonia** | **Meningitis** | **Inpatient NPNM** | **Cases Averted** | **Deaths Averted** |
| Anhui | 564 | 2647 | 61 | 21 | 7 | 25 | 3 | 0 | 3293 | 28 |
| Beijing | 147 | 688 | 7 | 2 | 1 | 3 | 0 | 0 | 844 | 4 |
| Chongqing | 135 | 633 | 10 | 3 | 1 | 5 | 0 | 0 | 782 | 5 |
| Fujian | 644 | 3024 | 51 | 17 | 6 | 24 | 2 | 0 | 3737 | 26 |
| Gansu | 1148 | 5389 | 310 | 105 | 36 | 116 | 14 | 0 | 6952 | 130 |
| Guangdong | 738 | 3464 | 48 | 16 | 6 | 23 | 2 | 0 | 4267 | 25 |
| Guangxi | 833 | 3908 | 71 | 24 | 8 | 40 | 3 | 0 | 4835 | 44 |
| Guizhou | 985 | 4624 | 73 | 25 | 8 | 57 | 3 | 0 | 5707 | 60 |
| Hainan | 187 | 875 | 32 | 11 | 4 | 23 | 1 | 0 | 1105 | 24 |
| Hebei | 2037 | 9559 | 423 | 143 | 49 | 114 | 19 | 0 | 12162 | 133 |
| Heilongjiang | 323 | 1515 | 43 | 14 | 5 | 12 | 2 | 0 | 1895 | 14 |
| Henan | 450 | 2110 | 65 | 22 | 8 | 20 | 3 | 0 | 2646 | 23 |
| Hubei | 200 | 940 | 12 | 4 | 1 | 7 | 1 | 0 | 1156 | 8 |
| Hunan | 1092 | 5125 | 48 | 16 | 6 | 29 | 2 | 0 | 6281 | 31 |
| Inner Mongolia | 817 | 3834 | 155 | 52 | 18 | 51 | 7 | 0 | 4858 | 58 |
| Jiangsu | 2093 | 9822 | 76 | 26 | 9 | 27 | 3 | 0 | 12017 | 31 |
| Jiangxi | 342 | 1603 | 73 | 24 | 8 | 37 | 3 | 0 | 2042 | 40 |
| Jilin | 505 | 2369 | 79 | 27 | 9 | 21 | 4 | 0 | 2979 | 25 |
| Liaoning | 738 | 3464 | 31 | 11 | 4 | 10 | 1 | 0 | 4244 | 11 |
| Ningxia | 314 | 1473 | 59 | 20 | 7 | 32 | 3 | 0 | 1865 | 35 |
| Qinghai | 307 | 1442 | 105 | 35 | 12 | 55 | 5 | 0 | 1889 | 60 |
| Shaanxi | 1064 | 4993 | 252 | 85 | 29 | 100 | 11 | 0 | 6394 | 111 |
| Shandong | 1008 | 4728 | 67 | 23 | 8 | 16 | 3 | 0 | 5826 | 19 |
| Shanghai | 51 | 237 | 2 | 1 | 0 | 1 | 0 | 0 | 291 | 1 |
| Shanxi | 1048 | 4919 | 230 | 78 | 27 | 84 | 10 | 0 | 6275 | 94 |
| Sichuan | 394 | 1848 | 40 | 13 | 5 | 28 | 2 | 0 | 2294 | 30 |
| Tianjin | 48 | 227 | 4 | 1 | 0 | 2 | 0 | 0 | 281 | 2 |
| Tibet | 291 | 1365 | 30 | 10 | 3 | 74 | 6 | 0 | 1695 | 80 |
| Xinjiang | 1474 | 6916 | 268 | 90 | 31 | 359 | 32 | 0 | 8748 | 391 |
| Yunnan | 1592 | 7471 | 239 | 81 | 28 | 201 | 11 | 0 | 9383 | 212 |
| Zhejiang | 288 | 1351 | 16 | 5 | 2 | 6 | 1 | 0 | 1660 | 7 |
| **East** | 7978 | 37441 | 758 | 256 | 88 | 250 | 34 | 0 | 46433 | 284 |
| **West** | 4523 | 21227 | 611 | 206 | 71 | 235 | 27 | 0 | 26568 | 263 |
| **Central** | 9353 | 43894 | 1612 | 543 | 187 | 1119 | 97 | 1 | 55402 | 1216 |
| **National** | 21855 | 102562 | 2981 | 1005 | 346 | 1604 | 157 | 1 | 128404 | 1763 |

Rows and columns may not sum to the total due to rounding.
