# Supplementary material for: National and provincial impact and cost-effectiveness of Haemophilus influenzae type b conjugate vaccine in China: a modeling analysis
Source: BMC Med. 2021 Aug 11;19:181. doi: 10.1186/s12916-021-02049-7 (PMC8356460; doi:10.1186/s12916-021-02049-7)
Supplement: Supplementary file 7 — Additional file 7: Table S17- Incremental cost-effectiveness ratios (US$) for the different Hib vaccine schedules (4 doses vs. 3 doses in the NIP); Table S18- National incremental cost-effectiveness ratios (US$ per QALY gained) when varying the NIP Hib vaccine price; Table S19- Provincial incremental cost-effectiveness ratios (US$ per QALY gained) when reducing the NIP Hib vaccine price. [file 12916_2021_2049_MOESM7_ESM.docx]

**Additional file 7.** **Results of vaccine dosing schedule and vaccine price scenario sensitivity analyses**

$${VEC}_{combined}= \left\{ \begin{aligned} {VEC}_{direct} , &C<10\% or C\geq98\% \\ \left( C\times2.428 \right)-0.138, &10\% \leq C < 40\% \\ (C*2.428) - ((C-0.40)*2.257) - 0.138, &40\% \leq C < 98\% \end{aligned} \right.$$

*Vaccine dosing schedule sensitivity analysis*

**Table 1. Incremental cost-effectiveness ratios (US$) for the different Hib vaccine schedules (4 doses vs. 3 doses in the NIP)**

| **Province and Region** | **4 Dose Schedule** | | | | **3 Dose Schedule** | | | |
| --- | --- | --- | --- | --- | --- | --- | --- | --- |
|  | **Cost per Case Averted** | **Cost per Death Averted** | **Cost per QALY gained** | **Rank** | **Cost per Case Averted** | **Cost per Death Averted** | **Cost per QALY gained** | **Rank** |
| Anhui | 3,473 | 405,945 | 12,628 | **18** | 1,898 | 221,840 | 6,901 | **17** |
| Beijing | 3,660 | 842,025 | 26,151 | **26** | 1,958 | 450,439 | 13,989 | **27** |
| Chongqing | 3,045 | 442,970 | 13,866 | **19** | 1,525 | 221,876 | 6,945 | **19** |
| Fujian | 4,873 | 688,732 | 21,535 | **23** | 2,986 | 421,988 | 13,195 | **26** |
| Gansu | 1,263 | 67,502 | 2,109 | **5** | 418 | 22,340 | 698 | **8** |
| Guangdong | 3,196 | 542,026 | 16,881 | **22** | 1,527 | 259,026 | 8,067 | **21** |
| Guangxi | 4,059 | 450,407 | 14,269 | **20** | 2,531 | 280,809 | 8,896 | **23** |
| Guizhou | 3,906 | 370,831 | 11,968 | **16** | 2,454 | 233,026 | 7,520 | **20** |
| Hainan | 1,840 | 84,335 | 2,725 | **6** | 385 | 17,658 | 570 | **6** |
| Hebei | 1,775 | 161,982 | 4,892 | **12** | 780 | 71,164 | 2,149 | **14** |
| Heilongjiang | 2,743 | 378,325 | 11,515 | **15** | 1,649 | 227,508 | 6,925 | **18** |
| Henan | 4,362 | 508,959 | 15,469 | **21** | 2,298 | 268,097 | 8,148 | **22** |
| Hubei | 4,459 | 681,881 | 21,623 | **24** | 2,281 | 348,860 | 11,062 | **24** |
| Hunan | 4,770 | 956,977 | 30,066 | **28** | 3,067 | 615,333 | 19,332 | **28** |
| Inner Mongolia | 1,403 | 117,771 | 3,620 | **9** | 636 | 53,390 | 1,641 | **10** |
| Jiangsu | 4,391 | 1,709,314 | 50,853 | **31** | 2,971 | 1,156,471 | 34,406 | **31** |
| Jiangxi | 1,811 | 92,556 | 2,938 | **7** | 393 | 20,087 | 638 | **7** |
| Jilin | 1,457 | 174,082 | 5,231 | **14** | 720 | 86,079 | 2,586 | **15** |
| Liaoning | 3,236 | 1,233,132 | 36,353 | **29** | 2,169 | 826,558 | 24,367 | **29** |
| Ningxia | 1,806 | 96,869 | 3,091 | **8** | 863 | 46,313 | 1,478 | **9** |
| Qinghai | Cost-saving | Cost-saving | Cost-saving | **3** | Cost-saving | Cost-saving | Cost-saving | **3** |
| Shaanxi | 2,148 | 123,326 | 3,857 | **11** | 1,020 | 58,592 | 1,832 | **13** |
| Shandong | 5,555 | 1,687,784 | 49,231 | **30** | 3,473 | 1,055,348 | 30,783 | **30** |
| Shanghai | 3,506 | 776,871 | 24,137 | **25** | Cost-saving | Cost-saving | Cost-saving | **5** |
| Shanxi | 1,807 | 120,112 | 3,726 | **10** | 884 | 58,771 | 1,823 | **12** |
| Sichuan | 2,110 | 160,170 | 5,157 | **13** | 704 | 53,490 | 1,722 | **11** |
| Tianjin | 2,885 | 391,035 | 12,278 | **17** | 1,019 | 138,076 | 4,336 | **16** |
| Tibet | Cost-saving | Cost-saving | Cost-saving | **1** | Cost-saving | Cost-saving | Cost-saving | **1** |
| Xinjiang | Cost-saving | Cost-saving | Cost-saving | **2** | Cost-saving | Cost-saving | Cost-saving | **2** |
| Yunnan | 497 | 22,055 | 719 | **4** | Cost-saving | Cost-saving | Cost-saving | **4** |
| Zhejiang | 3,428 | 872,003 | 26,459 | **27** | 1,640 | 417,245 | 12,661 | **25** |
| **East** | 3,583 | 600,809 | 18,369 |  | 1,997 | 334,872 | 10,238 |  |
| **West** | 3,396 | 348,048 | 10,815 |  | 1,821 | 186,695 | 5,801 |  |
| **Central** | 1,722 | 89,674 | 2,898 |  | 602 | 31,325 | 1,012 |  |
| **National** | 2,899 | 252,686 | 8,001 |  | 1,475 | 128,562 | 4,071 |  |

^*^The 4 dose schedule was used in the base case analysis.

*Vaccine price sensitivity analysis*

To estimate the influence of vaccine price in the NIP on cost-effectiveness, we reduced the price of a Hib vaccine dose by 10%, 25%, 50%, 75% at national level. The results of the vaccine price sensitivity analysis are in Table 2.

**Table 2. National incremental cost-effectiveness ratios (US$ per QALY gained) when varying the NIP Hib vaccine price**

| **NIP Hib Vaccine Price per dose (US$)** | **Cost per QALY gained**  **(without herd immunity)** | **Cost per QALY gained**  **(with herd immunity)** |
| --- | --- | --- |
| 11.62 (base case) | 8,001 | 14,850 |
| 10.46 (10% reduction) | 7,032 | 13,409 |
| 8.72 (25% reduction) | 5,578 | 11,167 |
| 5.81 (50% reduction) | 3,155 | 7,431 |
| 2.91 (75% reduction) | 732 | 3,695 |

**Table 3. Provincial incremental cost-effectiveness ratios (US$ per QALY gained) when reducing the NIP Hib vaccine price**

| **Province and Region** | **Cost per QALY gained (RMB)** | | | | | **Cost per QALY gained (US$)** | | | | |
| --- | --- | --- | --- | --- | --- | --- | --- | --- | --- | --- |
|  | **Base Case** | **10% Decrease** | **25% Decrease** | **50% Decrease** | **75% Decrease** | **Base Case** | **10% Decrease** | **25% Decrease** | **50% Decrease** | **75% Decrease** |
| Anhui | 85,870 | 75,609 | 60,219 | 34,568 | 8,917 | 12,628 | 11,119 | 8,856 | 5,083 | 1,311 |
| Beijing | 177,827 | 161,800 | 137,758 | 97,688 | 57,619 | 26,151 | 23,794 | 20,258 | 14,366 | 8,473 |
| Chongqing | 94,291 | 82,324 | 64,373 | 34,454 | 4,535 | 13,866 | 12,106 | 9,467 | 5,067 | 667 |
| Fujian | 146,437 | 133,718 | 114,638 | 82,838 | 51,039 | 21,535 | 19,664 | 16,859 | 12,182 | 7,506 |
| Gansu | 14,339 | 11,695 | 7,729 | 1,118 | Cost-Saving | 2,109 | 1,720 | 1,137 | 164 | Cost-Saving |
| Guangdong | 114,793 | 100,392 | 78,789 | 42,785 | 6,781 | 16,881 | 14,764 | 11,587 | 6,292 | 997 |
| Guangxi | 97,031 | 88,420 | 75,503 | 53,975 | 32,447 | 14,269 | 13,003 | 11,103 | 7,938 | 4,772 |
| Guizhou | 81,380 | 73,649 | 62,053 | 42,726 | 23,399 | 11,968 | 10,831 | 9,125 | 6,283 | 3,441 |
| Hainan | 18,528 | 15,242 | 10,314 | 2,101 | Cost-Saving | 2,725 | 2,242 | 1,517 | 309 | Cost-Saving |
| Hebei | 33,263 | 28,149 | 20,479 | 7,695 | Cost-Saving | 4,892 | 4,140 | 3,012 | 1,132 | Cost-Saving |
| Heilongjiang | 78,303 | 71,191 | 60,522 | 42,741 | 24,960 | 11,515 | 10,469 | 8,900 | 6,285 | 3,671 |
| Henan | 105,186 | 92,263 | 72,879 | 40,571 | 8,263 | 15,469 | 13,568 | 10,717 | 5,966 | 1,215 |
| Hubei | 147,034 | 128,391 | 100,428 | 53,822 | 7,216 | 21,623 | 18,881 | 14,769 | 7,915 | 1,061 |
| Hunan | 204,445 | 186,242 | 158,936 | 113,427 | 67,918 | 30,066 | 27,389 | 23,373 | 16,680 | 9,988 |
| Inner Mongolia | 24,619 | 21,178 | 16,018 | 7,417 | Cost-Saving | 3,620 | 3,114 | 2,356 | 1,091 | Cost-Saving |
| Jiangsu | 345,802 | 321,793 | 285,780 | 225,759 | 165,737 | 50,853 | 47,322 | 42,026 | 33,200 | 24,373 |
| Jiangxi | 19,978 | 15,772 | 9,463 | Cost-Saving | Cost-Saving | 2,938 | 2,319 | 1,392 | Cost-Saving | Cost-Saving |
| Jilin | 35,569 | 30,901 | 23,899 | 12,229 | 559 | 5,231 | 4,544 | 3,515 | 1,798 | 82 |
| Liaoning | 247,203 | 228,924 | 201,504 | 155,805 | 110,106 | 36,353 | 33,665 | 29,633 | 22,912 | 16,192 |
| Ningxia | 21,016 | 18,212 | 14,007 | 6,997 | Cost-Saving | 3,091 | 2,678 | 2,060 | 1,029 | Cost-Saving |
| Qinghai | Cost-Saving | Cost-Saving | Cost-Saving | Cost-Saving | Cost-Saving | Cost-Saving | Cost-Saving | Cost-Saving | Cost-Saving | Cost-Saving |
| Shaanxi | 26,227 | 22,708 | 17,429 | 8,631 | Cost-Saving | 3,857 | 3,339 | 2,563 | 1,269 | Cost-Saving |
| Shandong | 334,769 | 302,539 | 254,193 | 173,617 | 93,041 | 49,231 | 44,491 | 37,381 | 25,532 | 13,682 |
| Shanghai | 164,129 | 126,846 | 70,920 | Cost-Saving | Cost-Saving | 24,137 | 18,654 | 10,429 | Cost-Saving | Cost-Saving |
| Shanxi | 25,339 | 21,729 | 16,314 | 7,288 | Cost-Saving | 3,726 | 3,195 | 2,399 | 1,072 | Cost-Saving |
| Sichuan | 35,069 | 28,992 | 19,877 | 4,685 | Cost-Saving | 5,157 | 4,264 | 2,923 | 689 | Cost-Saving |
| Tianjin | 83,493 | 71,379 | 53,209 | 22,925 | Cost-Saving | 12,278 | 10,497 | 7,825 | 3,371 | Cost-Saving |
| Tibet | Cost-Saving | Cost-Saving | Cost-Saving | Cost-Saving | Cost-Saving | Cost-Saving | Cost-Saving | Cost-Saving | Cost-Saving | Cost-Saving |
| Xinjiang | Cost-Saving | Cost-Saving | Cost-Saving | Cost-Saving | Cost-Saving | Cost-Saving | Cost-Saving | Cost-Saving | Cost-Saving | Cost-Saving |
| Yunnan | 4,887 | 2,728 | Cost-Saving | Cost-Saving | Cost-Saving | 719 | 401 | Cost-Saving | Cost-Saving | Cost-Saving |
| Zhejiang | 179,924 | 158,704 | 126,875 | 73,826 | 20,777 | 26,459 | 23,339 | 18,658 | 10,857 | 3,055 |
| East | 124,908 | 111,755 | 92,025 | 59,142 | 26,259 | 18,369 | 16,434 | 13,533 | 8,697 | 3,862 |
| West | 19,708 | 16,448 | 11,558 | 3,408 | Cost-Saving | 2,898 | 2,419 | 1,700 | 501 | Cost-Saving |
| Central | 73,540 | 64,702 | 51,445 | 29,350 | 7,256 | 10,815 | 9,515 | 7,565 | 4,316 | 1,067 |
| National | 54,406 | 47,766 | 37,806 | 21,206 | 4,607 | 8,001 | 7,024 | 5,560 | 3,119 | 677 |
